# Expert Perspective: Who May Benefit Most From the New Ultra Long-Term Subcutaneous EEG Monitoring?

**DOI:** 10.3389/fneur.2021.817733

**Published:** 2022-01-20

**Authors:** Jay Pathmanathan, Troels W. Kjaer, Andrew J. Cole, Norman Delanty, Rainer Surges, Jonas Duun-Henriksen

**Affiliations:** ^1^Department of Neurology, Perelman School of Medicine, University of Pennsylvania, Philadelphia, PA, United States; ^2^Department of Neurology, Center of Neurophysiology, Zealand University Hospital, Roskilde, Denmark; ^3^Department of Clinical Medicine, University of Copenhagen, Copenhagen, Denmark; ^4^Massachusetts General Hospital and Harvard Medical School, Boston, MA, United States; ^5^Department of Neurology, Beaumont Hospital, Dublin, Ireland; ^6^FutureNeuro Research Centre, Dublin, Ireland; ^7^School of Pharmacy and Biomolecular Sciences, Royal College of Surgeons in Ireland, Dublin, Ireland; ^8^Department of Epileptology, University Hospital Bonn, Bonn, Germany; ^9^UNEEG medical, Alleroed, Denmark

**Keywords:** epilepsy monitoring and recording, seizure detection, circadian rhythm, chronotherapy, subcutaneous EEG, sub-scalp

## Abstract

Today's modalities for short-term monitoring of EEG are primarily meant for supporting clinical diagnosis of epilepsy or classifying seizures and interictal epileptiform discharges while long-term EEG adds the value of differential diagnosis investigation or pre-surgical evaluation. However, longitudinal epilepsy care relies on patient diaries, which is known to be unreliable for most patients and especially those with focal impaired awareness or nocturnal seizures. The subcutaneous ultra long-term EEG (ULT-EEG) systems alleviate those issue by enabling objective, continuous EEG monitoring for days, weeks, months, or years. Albeit a great advance in continuous EEG over extended periods, it comes with the caveat of limited spatial resolution of two channels. Therefore, the new subcutaneous EEG modality may be especially suited for a selected group of patients. We convened a panel of experienced epileptologists to consider the utility of a subcutaneous, two-channel ULT-EEG device with the goal of developing a consensus-based expert recommendation on selecting the optimal patient types for this investigative technique. The ideal patients to select for this type of monitoring would have focal impaired awareness seizures without predominant motor features and seizures with medium to high voltage patterns. As this technology matures and we learn more about its limitations and benefits we might find a wider array of use case scenarios as it is believed that the benefits for many patients are most likely to outweigh the risks and cost.

## Introduction

The clinical use of human electroencephalography (EEG) is approaching its 100th anniversary. Technical advances in computational power, hardware size and power requirements, data storage, and network bandwidth have resulted in digitization of data and in large growth of the use of inpatient continuous EEG (1) as well as an increase in the use of 1–3 days ambulatory recordings. However, in terms of using the EEG for ultra long-term monitoring as done with electrocardiography in cardiac diseases, no notable advances have taken place until recently.

In 2019, the first device for subcutaneous ULT-EEG recording was marketed in Europe, and multiple other devices are also in development (2). In the current paper we discuss limitations in current practice, relate it to patients for whom ULT-EEG recordings would be beneficial, and identify situations in which they would be unlikely to have significant utility. We convened a panel of experienced epileptologists to consider the utility of ULT-EEG with the goal of developing a consensus-based expert recommendation. Three patients from a trial with the marketed device (3) were presented; first as a standard-of-care case, and then added with the extra information the subcutaneous device provided (clinical vignettes and subcutaneous EEG reports available as Supplementary Material). The three cases were selected based on their diversity. One patient registered no seizures in her diary but presented 16 electrographic seizures during the trial consisting of both tonic-clonic and focal impaired awareness seizures. One patient registered seizures approximately on same days as electrographic focal seizures were identified, and one patient registered a lot more than what could be identified in the subcutaneous EEG although not correlated to those actually found (3). The cases were discussed in general terms leading to the different patient-types identified in this report.

### Limitations in Current Practice

Seizures are rather stochastic events, and the inter-event interval can range from minutes to years. EEGs, in part because of the limitations of current technology, are typically recorded for hours or days (4). To capture events, one typically needs to record for at least half the inter-event interval to have a 50% chance of success (2). Additionally, because most EEGs are performed in a medical setting, the cerebral response to normal stressors experienced in daily life may not be replicated in a monitored and artificial setting (5). Because clinical events are difficult to capture, clinicians rely heavily on interictal abnormalities including slowing, spikes, and response to provocative stimuli to make inferences regarding the presence and nature of epileptic seizures in individual patients.

For those reasons, the intermittent use of standard EEG recording is often limited in the management of epilepsy. The more than 99% of time spent between seizures (the interictal period) is therefore often used to look for biomarkers as a surrogate for ictal activity with the presence of interictal epileptiform discharges (IEDs) used to infer the diagnosis of epilepsy, the risk of future seizures, and especially in genetic generalized epilepsies; the response to antiseizure medication (ASM) (6). IEDs are highly specific for an epilepsy diagnosis, which makes the association valid, but in other cases the association between IEDs and seizure frequency or severity is at best variable (7). Furthermore, the absence of an epileptiform discharge cannot rule out a diagnosis of epilepsy (6). Neurologists still lack the basic “loop recorder” used by cardiology for 3 decades and are left resorting to “divining” insight from transients seen on limited data sets. However, as opposed to long-term electrocardiography where amplitudes are measured in millivolts, EEG amplitudes recorded from the scalp are measured in microvolts. Because of this large order of magnitude difference, maintenance of noise-free scalp EEG over prolonged periods is difficult if not impossible, especially in naturalistic environments. On top of that, patients are often reluctant to wear EEG electrodes on their heads in their everyday environment due to the stigmatization.

Longitudinal epilepsy care is complicated by poor patient reporting. Half of patients with drug resistant epilepsy have a very difficult time keeping a reliable diary (8) and patients with focal impaired awareness or nocturnal seizures might only recognize 30% or less of their seizures. Thus, taken together, there is a substantial deficit when the treating neurologist relies on patient self-reporting, and grossly limited EEG recordings to optimize therapy.

There are now some seizure detection devices with medical device approval in both United States and Europe that can aid in seizure counting. Wearables for tonic-clonic seizures have demonstrated validity and usefulness in daily life but are not of use for patients with non-motor seizures (9). For a small subset of drug resistant patients in the USA who were not deemed candidates for resective surgical therapy, the NeuroPace RNS^®^ system may be implanted with the goal of reducing seizure frequency through closed loop stimulation (10). Some limited ECoG data may be available along with seizure count, but it is not possible to verify whether all event detections are true seizures, subclinical interictal runs, or artifacts (11). Neither is it possible to investigate for missing detections as data is not logged continuously. For patients with bilateral seizures using the responsive neurostimulation device the average time to record bilateral electrographic seizures was 41.6 days, which shows that they can be difficult to record in an in-hospital long-term recording usually lasting for 1–2 weeks (12).

### Subcutaneous Ultra Long-Term EEG as a Modality

**Figure 1:**
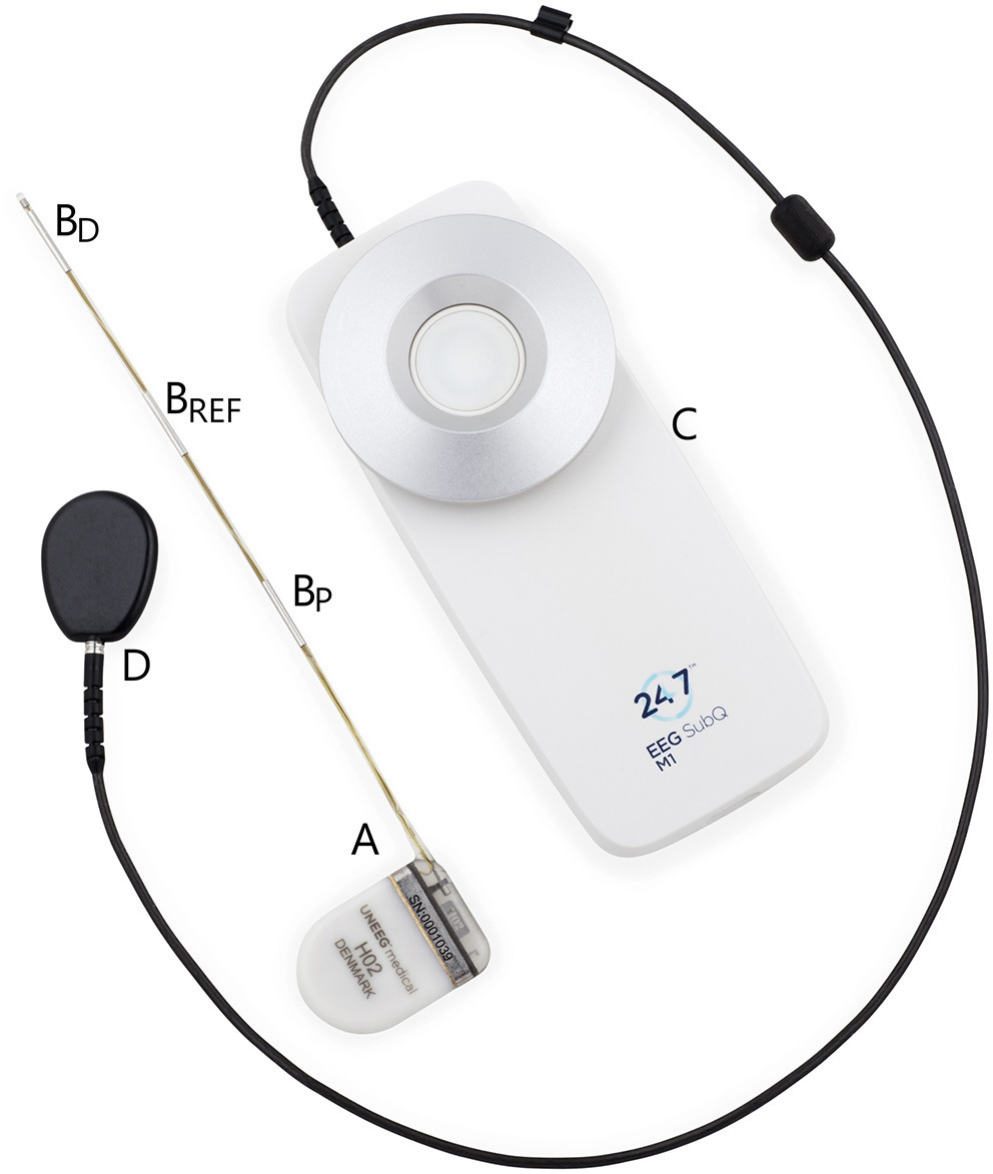
The two physical parts of the discussed ultra long-term subcutaneous EEG recorder. The implantable part (A) is placed extracranially underneath the skin. It measures bipolar EEG from the contact points (B_D_-B_REF_ and B_P_-B_REF_) with the center electrode as the reference and an interelectrode distance of 3.5 cm. The external part (C) is attached to the clothes of the person with epilepsy and the disk (D) is attached to the skin aligned with the housing of the implant (A). As soon as the external disk and the implant housing are aligned, EEG is recorded and stored.

The subcutaneous ULT-EEG recorder discussed in the consensus-meeting was the 24/7*EEG*™ *SubQ* from *UNEEG medical* (Alleroed, Denmark) as seen in Figure 1. The device consists of two parts; an implant with three electrodes and an external storage unit that also powers up the implant through an inductive link. When the two parts are aligned on opposite sides of the skin, EEG is recorded in two bipolar channels with a sampling frequency of 207 Hz. More details can be found in Duun-Henriksen et al. (2).

The implant can be inserted in a minimally invasive procedure under local anesthesia with locations over most of the head. In the cases discussed it had a horizontal direction over the temporal lobe toward the temple as seen in Weisdorf et al. (13). The house of the external device is worn on the clothes of the patient. It needs to be changed daily, so that one device is charging, and one is recording. The patient can insert a seizure marker in data by pressing a button, but apart from this, no interaction is needed by the user.

Subcutaneous EEG demonstrates identical ictal and interictal patterns to scalp EEG at similar locations (13, 14). The major advantage is longevity as it maintains the electrographic characteristics with no human intervention, allowing for stable EEG over timescales of many months or even years. However, the discussed subcutaneous EEG solution is restricted to unilateral recording over a limited spatial area (outermost electrodes 7 cm apart). Therefore, a priori understanding of the patient's seizure morphology and localization is essential as the placement of the electrodes would need to be selected based on a known or suspected epileptic focus identified by either clinical semiology, prior EEG abnormalities, or an identified structural lesion on brain imaging in a patient with unequivocal epilepsy.

## Patient Groups

### The Patient With Largest Promise

Based on the limitations of the described device, the discussants universally agreed that the patients who may benefit most by receiving the debated implanted subcutaneous monitor would be those who suffer from unequivocal drug resistant seizures with clear singular focus electrographic patterns, and who are unaware of many of their events either due to retrograde amnesia or nocturnal seizures. Given that the recording capabilities of the specific subcutaneous device are spatially limited, the patient's seizures would have to be well characterized prior to an implantation. Based on the data provided for review, it is likely that such a patient's seizures would be reliably captured—allowing for rapid medication adjustments by establishing the electrographic seizure burden and its correlation to symptoms and time of day with reliable outcome measures such as seizure frequency change and duration of seizures over time.

This type of recording is particularly valuable for patients with infrequent seizures, where a routine EEG or ambulatory EEG would be unlikely to capture an event, and in whom ASM withdrawal in the EMU would not offer data reflective of their daily experience when on medication. It is likely that some patients have unaware convulsive seizures, and in such patients this data might indicate the need for more aggressive therapy including earlier resective or ablative surgery. It would also be of interest to see circadian and multiday cycles of seizures—patterns that might warrant a change in medication timing or treatment paradigm and thus allow application of chrono-therapeutic concepts (15, 16) (see also EEG reports in Supplementary Material).

### The Patient With Expected Seizure Control

Another patient group would be those with confirmed epilepsy, and a known seizure focus, that is seemingly well controlled, but in whom signs of missed seizures (subjective cognitive impairment, unexplained bruising, nocturnal enuresis, etc.) are reported. If the true seizure rate is one per week the probability of recording a seizure in a 3-day EMU stay or during ambulatory recording is approximately 50% and this number drops quickly if seizures are less frequent (2). You might increase the chance of recording a seizure using provocative measures, such as tapering ASMs, but you would usually not do that if the epilepsy is reported to be controlled. It is also known that seizures are not uniformly distributed. This means that the chance drops further unless you will have some way of predicting a seizure cluster so that it coincides with the monitoring period (15). Based on its simultaneous video and broader spatial coverage, video-EEG is still superior in many situations to subcutaneous EEG but choosing the optimal admission period based on seizure periodicities remains a challenge.

### The Interesting Research Centered Trial

There are many unknowns in epilepsy semiology that could enable better treatment of patients if elucidated. The ULT-EEG provides data that is well understood, but has never been accessible in this longitudinal way. We believe that interrelations between various biomarkers will be better understood in the future when large databases start to emerge. Below are discussed three interesting research subjects.

One of the often-described interrelations is the one between sleep and epilepsy. It is well established that lack of sleep can increase the risk of seizures, but apparently too much sleep can do the same. Little is known how reproduceable this is between patients and within same subject. It is also believed that nocturnal seizures often happen in the same sleep phase for the individual patient (17) and we know that newer ASMs can change the REM-sleep which could be a biomarker for the efficiency of a certain drug on that individual. This can now be confirmed with multiple nocturnal EEG recordings. It has already been shown that sleep stages can be identified in the subcutaneous EEG (18) enabling further research on how to improve the epilepsy management by better understanding the interrelationship between sleep and epilepsy.

Another type of events already mentioned are interictal epileptiform discharges (IEDs). Their presence aids in diagnosis and management of epilepsy, and helps to confirm a clinical diagnosis of epilepsy, defining the epilepsy syndrome, provides information that assists in planning treatment management, and helps to assess candidacy for epilepsy surgery. The interrelation between IEDs and seizures has long been identified, however, the value of tracing them continuously over time seems promising although still speculative (19, 20). With the possibility of measuring EEG over ultra long-term the relationship can be investigated in greater detail and potentially be valuable for all intractable patients. However, interrater agreement of IEDs between subjective expert-scorings as well as algorithms for automatic detection are becoming better but still have room for improvement (21).

Finally, the post-ictal state can be identified in the EEG as suppression of physiological rhythms. There is a correlation between a generalized suppression and the risk of SUDEP (22). Little is known what value continuous two-channel recording correlates to SUDEP, how the post-ictal suppression fluctuates over time, and whether this also alters the risk of SUDEP. This would especially be relevant for patients with tonic-clonic or nocturnal seizures where the risk of SUDEP is largest. Wearable devices reliably detect tonic-clonic seizures, but can only provide, at its best, an indirect measure of suppression rate (23). Further research in post-ictal suppression might identify new biomarkers that can elucidate risk factors for the individual patient over time.

### Patients Who Are Unlikely to Benefit

Conceptually, the two-channel ULT-EEG device could be used for a purely diagnostic purpose in selected patients (for example, patients with reported generalized convulsions on a monthly timescale), but the risk of an incorrect diagnostic result when only having two-channels is unknown and presumably too high to be used for this purpose. The device can rule-in epileptiform seizures, but it cannot necessarily rule them out.

Moreover, patients with extratemporal seizure focus such as frontal lobe, mesial parietal, medial occipital or basal parietal/occipital often have none or only subtle EEG changes interictally as well as ictally. These patients are therefore less optimal candidates for subcutaneous recording unless clear ictal patterns are identifiable.

The last patient groups where the benefit is presumably low is the one with very high frequency of seizures. Although the patient might miss many events, the epilepsy management would probably not change even with a more precise seizure count. Neither would a patient with infrequent seizures but low adherence to using the subcutaneous EEG recorder obtain high value as it is unknown whether seizures might occur while the device is not used.

## Discussion

Ultra long-term EEG monitoring is unequivocally a seminal concept in epilepsy care, and multiple implementations from a variety of companies are imminent (2). It is less clear how neurologists should use this new technology, at least in the first-generation implementation with its associated technical limitations in various forms. Several important questions remain to be answered; How sensitive and specific is the data from this new technology for the detection of electrographic seizures of a given patient? How does the different devices differentiate? Will patients accept the technology? But also, in the hospital setting, answers need to be made; How will the sheer volume of data be handled? And most importantly, is this cost and care effective and appropriate for a given patient?

In the current paper we have identified the optimal patient for at two-channel subcutaneous ULT-EEG device, but we would like to elaborate a bit further on different use cases. The hallmark of ULT-EEG is the possibility to get an objective count of electrographic seizures in the every-day life of patients with epilepsy. This can be valuable in various situations in the patient journey. The most obvious impact will be for uncontrolled patients where a more accurate identification of an actual or increased seizure frequency can lead to better treatment and a continuous evaluation be performed assessing whether a seizure free state is obtained.

Whether continued monitoring is necessary if a seizure free state is obtained is debatable. However, epilepsy is known to be dynamic, and seizures can recur which might not be acknowledged by the patient immediately. Especially for patients who had a subcutaneous device implanted to obtain seizure freedom, could leave the device in, and only restart monitoring in case of new suspicion of seizure recurrence or in case of ASM change.

If medical treatment is not an option for the patient, exacerbations of the epilepsy can be followed with objective counting of seizures. It could also be used to empower the patient by identifying any underlying periodicities or triggers.

In patients where no diagnosis of epilepsy has yet been made for rare convulsive events, and who have failed empiric ASM trials, ULT-EEG recording might still have value if motor seizures are suspected. The key would be that the diagnosis itself provides value for example by reducing SUDEP risk, improving quality of life, or reducing additional diagnostic testing. An ULT-EEG solution with higher spatial resolution is very desired for broader diagnostic applications.

It should be noted that the ULT-EEG monitors will provide huge amounts of data that will not be feasible to manually review. No publications have yet shown what sensitivities and false positive rates can be expected from automatic detection of seizures. The diaries have sensitivities in the range of 30–50% (8) and thus the subcutaneous ULT-EEG should be better than this, and with quite a margin as the patients need to go through the hazzle of implantation and wearing a device. The potential false negative registrations include those from a focus not measurable at the implant location, an non-perfect algorithm including due to seizures not being visible in the two-channel recordings due to artifacts during e.g., motor seizures, and device down time either due to malfunctioning or the user not wearing the external part.

The use of this technology could also play an important role in trials of novel ASM. The current standard for identifying seizure burden—patient self-reporting—is known to be grossly inaccurate (8). In some patients, side effects, psychogenic events, and missed seizures could all confound trial data. In particular, drug trials focused on specific (and often severe) epileptic syndromes or genetic epilepsies would be relevant. Patients in these trials are most likely to have multiple seizure types and are often only documented by observers. Accurate seizure logging might even reduce the duration of each patient's enrollment, and thus lessen the cost of trials. However, if the patient group is heterogeneous, and only a subset can be enrolled to wear the subcutaneous EEG solution, it might complicate the trial.

**Figure 2:**
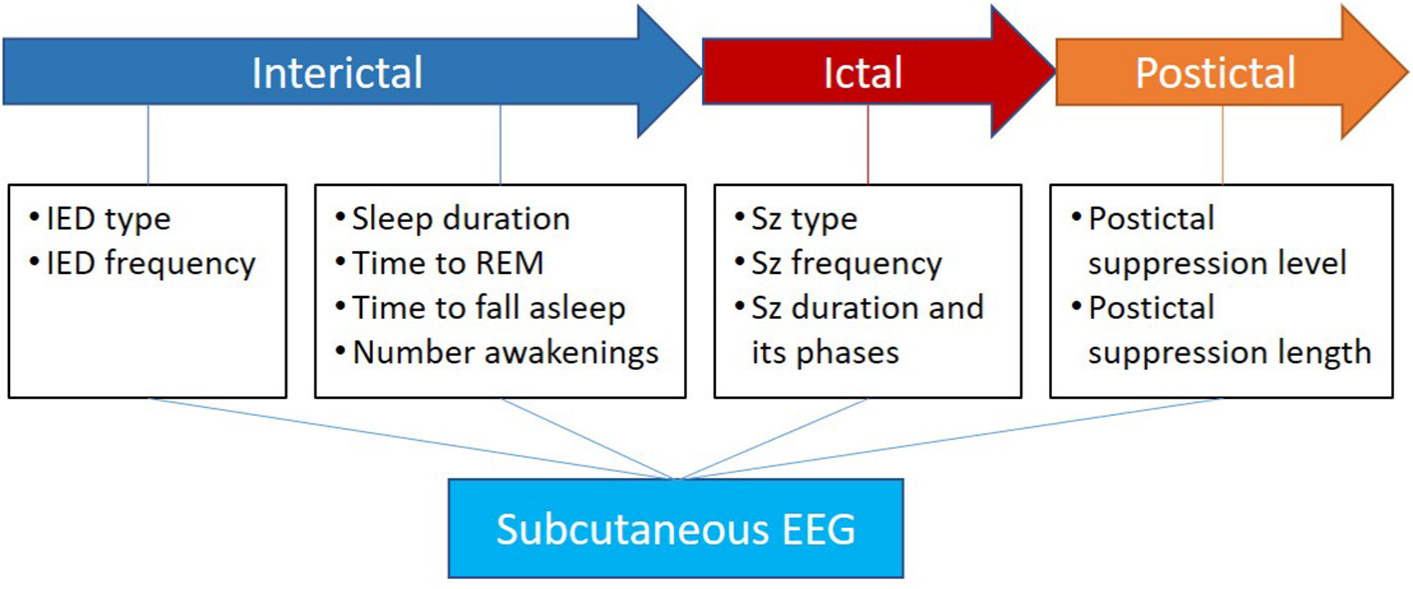
Apart from the direct value of ictal electrographic seizure description, the subcutaneous EEG has the potential to add valuable knowledge in the interictal phase about IEDs and sleep as well as the postictal phase about suppression level and length. Inspired by Beniczky et al. (24).

On top of objective seizure count and ictal EEG morphology, continuous ULT-EEG also provides data on post-ictal EEG findings including suppression level and duration. With adequate quantitative tools, ULT-EEG could also provide useful interictal and sleep data, as illustrated in Figure 2. Such parameters could prove useful to epilepsy care and in the evaluation of new compounds.

In contrast, the group felt that the device in focus was not necessarily the answer for one of the most difficult to diagnose patient populations in epilepsy: those with suspected psychogenic nonepileptic seizures (PNES). Because two-channel ULT-EEG is expected to have the same sensitivity as similarly placed scalp electrodes, some electrographic seizures are expected to be unrecordable with these recorders. PNES diagnosis continues to require multimodal data, in particular an assessment of EEG with semiology. Unfortunately, outside of self-described convulsive events, it is uncertain if a limited montage EEG data set would provide adequate answers. However, it is possible that future technological advancements with the primary addition of bilateral recording with better spatial coverage, but also addition of automatic detection of spikes, automatic sleep staging, and continuous recording without the need for patient intervention could make such technology more useful for the workup of PNES vs. electrographic seizures.

It has been established that the two-channel ULT-EEG system has a low spatial resolution to make the implantation procedure minimally invasive. With only two unilateral channels, patients with contralateral seizures could be misdiagnosed. It is therefore important to have a clear understanding whether the seizure morphology is visible at the location of the implant. Bilateral recording and potentially more electrodes would be a valuable addition for better spatial resolution.

Finally, some patients who would seem to be good candidates may not be. Although the implantation is minimally invasive it still introduces a foreign body in the sub-galeal space. This may be a contraindication in some patients, and in particular patients who might need to undergo cranial MR imaging in the near future, as the device, of present, is not MR compatible.

This first generation of subcutaneous devices has limitations but should be of value to a substantial subpopulation of the patients seen in the typical complex epilepsy clinic. At its present form, it will likely be restricted to the tertiary epilepsy clinic for selected patients—assuming the EEG interpretation is appropriately simplified and reimbursed. But regardless of the utility of this first-generation device, it is clear that we are on a path toward minimally invasive longer term outpatient monitoring for patients with with epilepsy and related disorders.

## Conclusion

The new subcutaneous ultra long-term EEG recorder considered in this paper is a step forward in epilepsy care, but there are potential pitfalls. Based on the discussion at this consensus meeting, the ideal patients to select for the two-channel ULT-EEG monitoring would have focal impaired awareness seizures without predominant motor features and seizures with medium to high voltage diffuse patterns. At present this reflects only a limited population of patients with epilepsy, but as this technology matures and we learn more about the limitations and benefits we might find a wider array of use case scenarios. However, drug resistant patients where there is a suspicion of unrecognized seizures or overreporting of events, should be considered for this type of monitoring while assessing the cost benefit to each patient. But with current limited evidence, it relies much on the discretion of the single neurologist. Ready or not, a new age in EEG recording has dawned.

## Data Availability Statement

The original contributions presented in the study are included in the article/supplementary material, further inquiries can be directed to the corresponding author.

## Ethics Statement

The studies involving human participants were reviewed and approved by Regional Science Ethics Committee of Zealand University Hospital (project no: SJ-551). The patients/participants provided their written informed consent to participate in this study. Written informed consent was obtained from the individual(s) for the publication of any potentially identifiable images or data included in this article.

## Author Contributions

JP, TK, and JD-H prepared data for the discussion and wrote the first draft of the manuscript. All authors participated in the roundtable discussion and contributed to the review of the manuscript.

## Conflict of Interest

All authors received a consultancy fee from UNEEG medical for the panel discussion. JP and TK consults for UNEEG medical. AC has received support from, and/or has served as a paid consultant for NeuroPace, Sage Therapeutics, and BrainVital/Precisis. ND has served on advisory boards and has received consultancy fees from UNEEG medical, Arvelle Therapeutics, Eisai, Sanofi, and UCB Pharma. RS reports lecture and consultancy fees from Angelini, Arvelle, Bial, Desitin, Eisai, LivaNova, Novartis, UCB Pharma, and UNEEG medical. JD-H is a full-time employee at UNEEG medical.

## Publisher's Note

All claims expressed in this article are solely those of the authors and do not necessarily represent those of their affiliated organizations, or those of the publisher, the editors and the reviewers. Any product that may be evaluated in this article, or claim that may be made by its manufacturer, is not guaranteed or endorsed by the publisher.
