# Supplementary material for: Expert Perspective: Who May Benefit Most From the New Ultra Long-Term Subcutaneous EEG Monitoring?
Source: Front Neurol. 2022 Jan 20;12:817733. doi: 10.3389/fneur.2021.817733 (PMC8810530; doi:10.3389/fneur.2021.817733)

**Clinical vignette 3 - SBN**

SBN experienced definite onset of seizures at age 18, although with suspicious depersonalization episodes from early childhood.

SBN described three types of seizures:

1. Focal aware seizure. These seizures included a sense of becoming someone else or being taken over, but with preserved awareness. Occasionally, the patient also experienced auditory distortions with voices being perceived as very high- or low-pitched, and rarely with visual distortions. These events were reported at 5-15 per day, mostly in the afternoon. SBN was uncertain about nocturnal events.
2. Type 1 seizures progressing to impaired awareness accompanied by oral automatisms. SBN had no recollection of this type of seizure.
3. Type 1 or 2 seizures progressing into BTCS. She reported 1-5 of these per month

Her neurological examination showed substantial impairment of short term memory. MRI (1.5T + 3T) showed no abnormalities. FDG-PET showed slightly reduced metabolic activity in the left frontal operculum/insula and lateral temporal cortex.

Two days of video-EEG (25 channels) revealed 24 electrographic seizures of types 1 and 2, characterized by 4 Hz activity with frequent sharp-waves in the left pre-, mid- and post-temporal areas including low-row (sub-temporal).

SBN attempted numerous antiepileptic drugs without obtaining seizure control:

1. Lamotrigine (ineffective as monotherapy)
2. Valproate (adverse effects: weight gain)
3. Lacosamide (increased seizure frequency)
4. Carbamazepine (adverse effects: dizziness)
5. Levetiracetam (increased seizure frequency and adverse effects: emotional lability)
6. Perampanel (side effects: pelvic pain)

SBN was referred to epilepsy surgery evaluation. At this point she was treated with lamotrigine 450 mg/day and brivaracetam 300 mg/day, and Zonisamide 200 mg/day. Repeat video-EEG (four days, 64 channels) revealed 21 seizures of type 1 and 2, with similar findings to the first video-EEG. Source localization suggested a posterior temporal focus.

**Seizure diary chart during study**


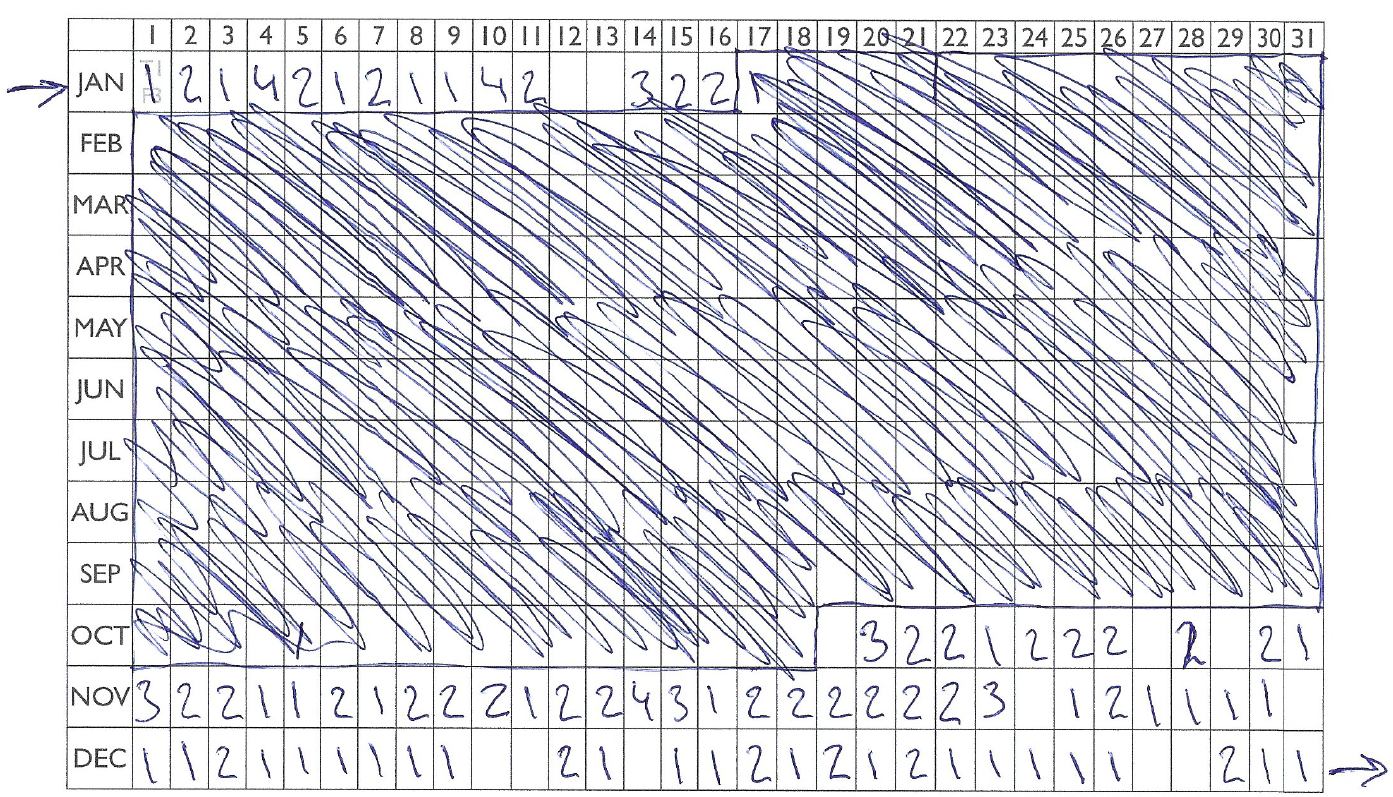

Supplement: Supplementary file 1 [file Data_Sheet_1.DOCX]
