# Supplementary material for: Expert Perspective: Who May Benefit Most From the New Ultra Long-Term Subcutaneous EEG Monitoring?
Source: Front Neurol. 2022 Jan 20;12:817733. doi: 10.3389/fneur.2021.817733 (PMC8810530; doi:10.3389/fneur.2021.817733)

**Clinical vignette 1 - J**

J experienced seizure onset at age 18, described only as complex partial seizures. An interictal EEG showed left temporal spike. MRI showed no abnormalities. Seizures were controlled with lamotrigine monotherapy for several years.

Treatment was discontinued, and J remained seizure free until age 42, at which time she began experiencing three types of seizures:

1. Focal onset seizure with impaired awareness, involving behavioral arrest and manual automatisms. She was amnestic to these events, which lasted 30-60 seconds.
2. Focal onset seizure with only impaired awareness, involving behavioral slowing but NOT arrest. During this time she was described as behaving inappropriately. She was amnestic to the majority of these events.
3. Type 1 or 2 seizure with progression to bilateral tonic-clonic seizure (BTCS).

J was unaware of many of her seizures, which were instead reported by family of colleagues. The self-reported seizure frequency was 2-4 seizures/year, but with at least 6-12 recognized by family/colleagues. Review of epilepsy symptoms only reviewed poor sleep, including awakening feeling unrefreshed.

Her neurological examination was normal, although she complained about increasing memory loss. Repeat brain MRI (1.5T) showed an old lacunar infarct (1 cm^3^) in the anterior of the left external capsule, but no abnormalities in the left temporal region. Due to the discrepancy between self-reported and observed seizures, the patient underwent repeated admissions to the epilepsy monitoring unit (each up to four days), where left frontotemporal seizures were captured after medication withdrawal and sleep deprivation.

MRI (1.5T, T1 without contrast) of old lacunar infarct (1 cm2)


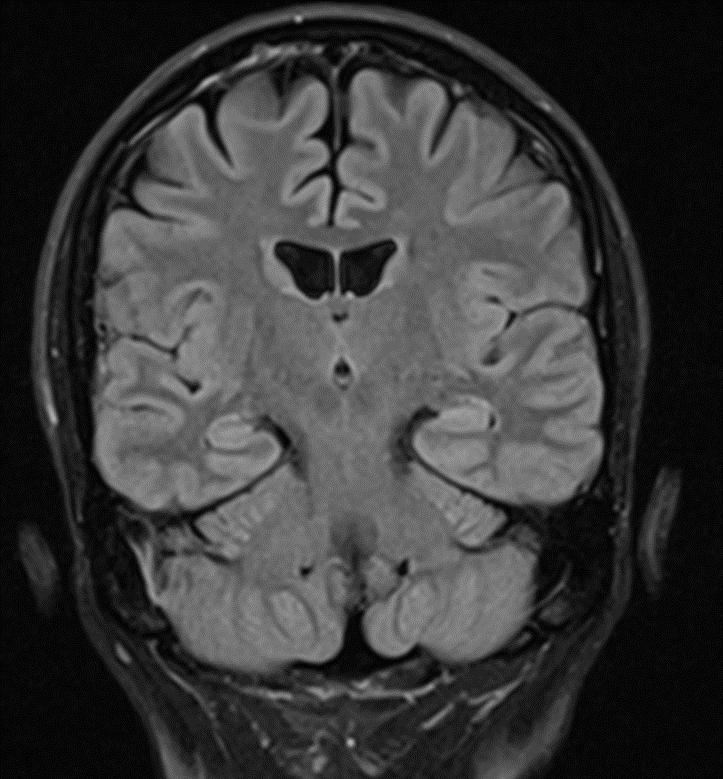

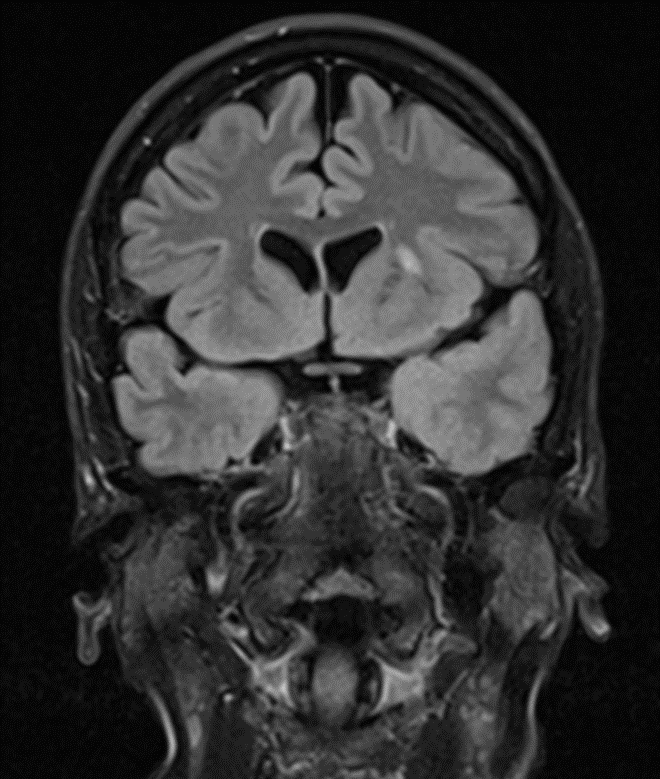

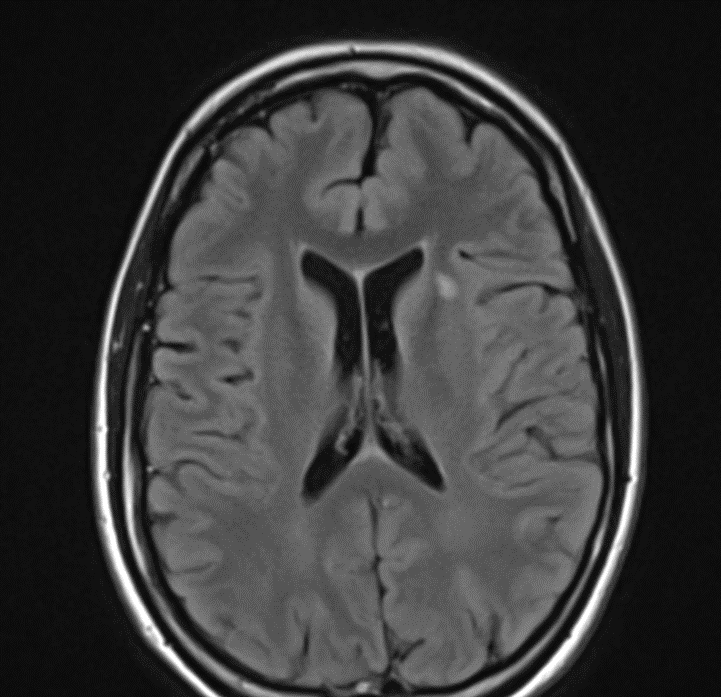


Treatment with several antiepileptic agents was attempted, including valproate up to 1500 mg/day and levetiracetam up to 2000 mg /day. Current treatment is a combination of lacosamide 400 mg/day and lamotrigine which was upregulated to 150 mg/day during the study period. She now reports being uncertain about her degree of control, but probably seizure frequency of 1-3 per month.

Focal to bilateral tonic-clonic seizure as recorded in the EMU with common average reference. First picture shows beginning of seizure. Second picture shows entire seizure.
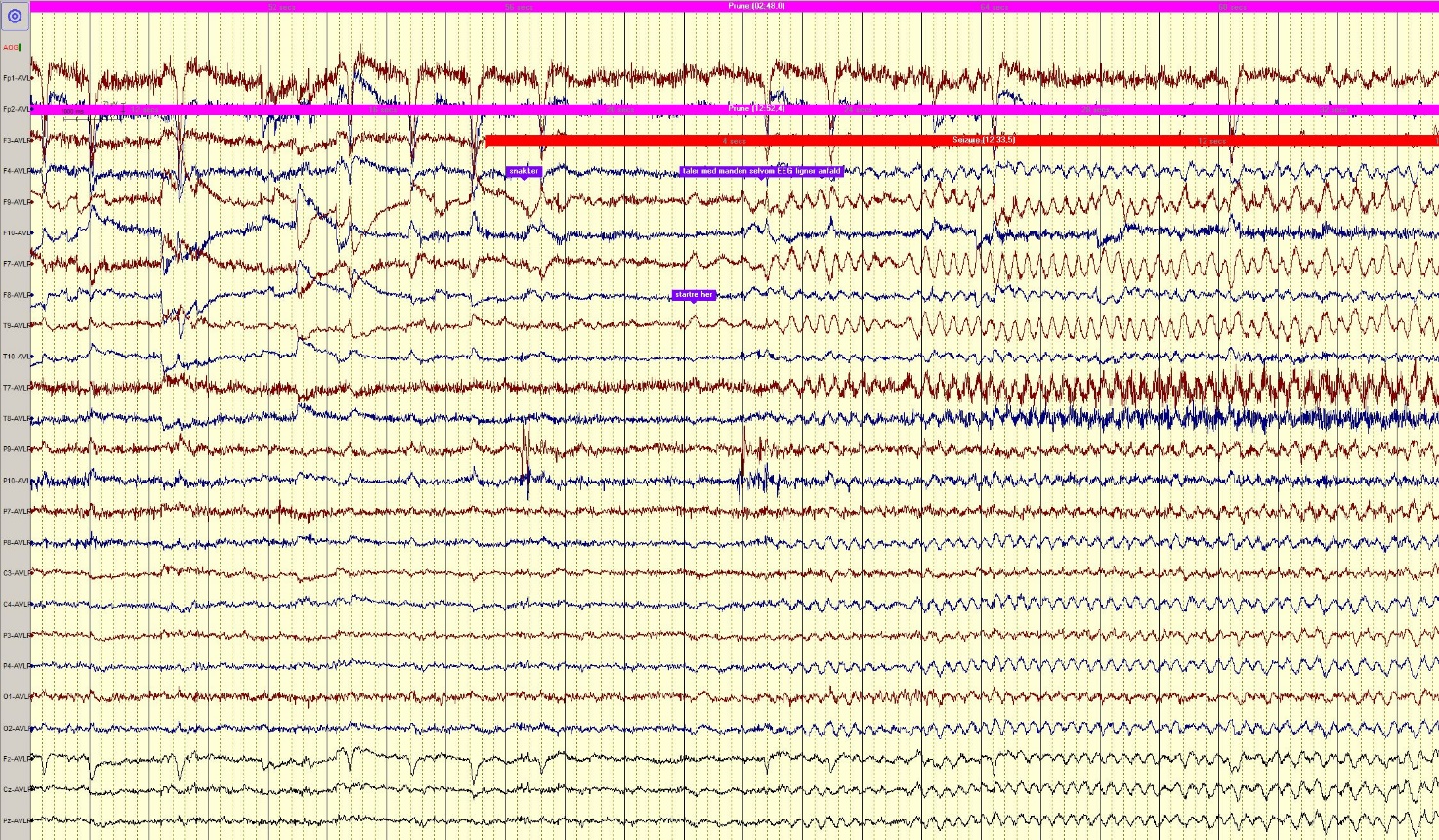

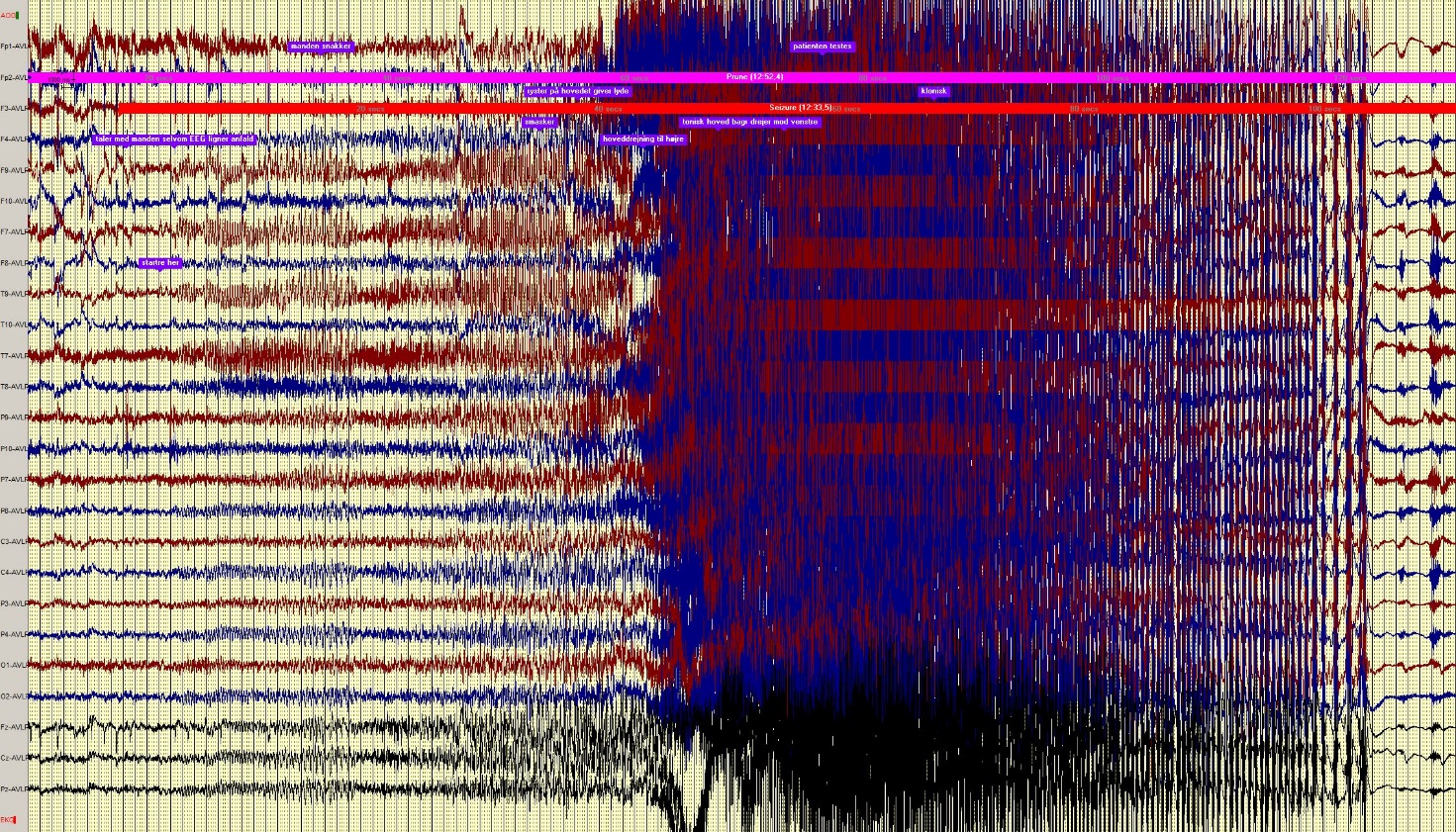
Focal Impaired Awareness seizure as recorded in EMU.
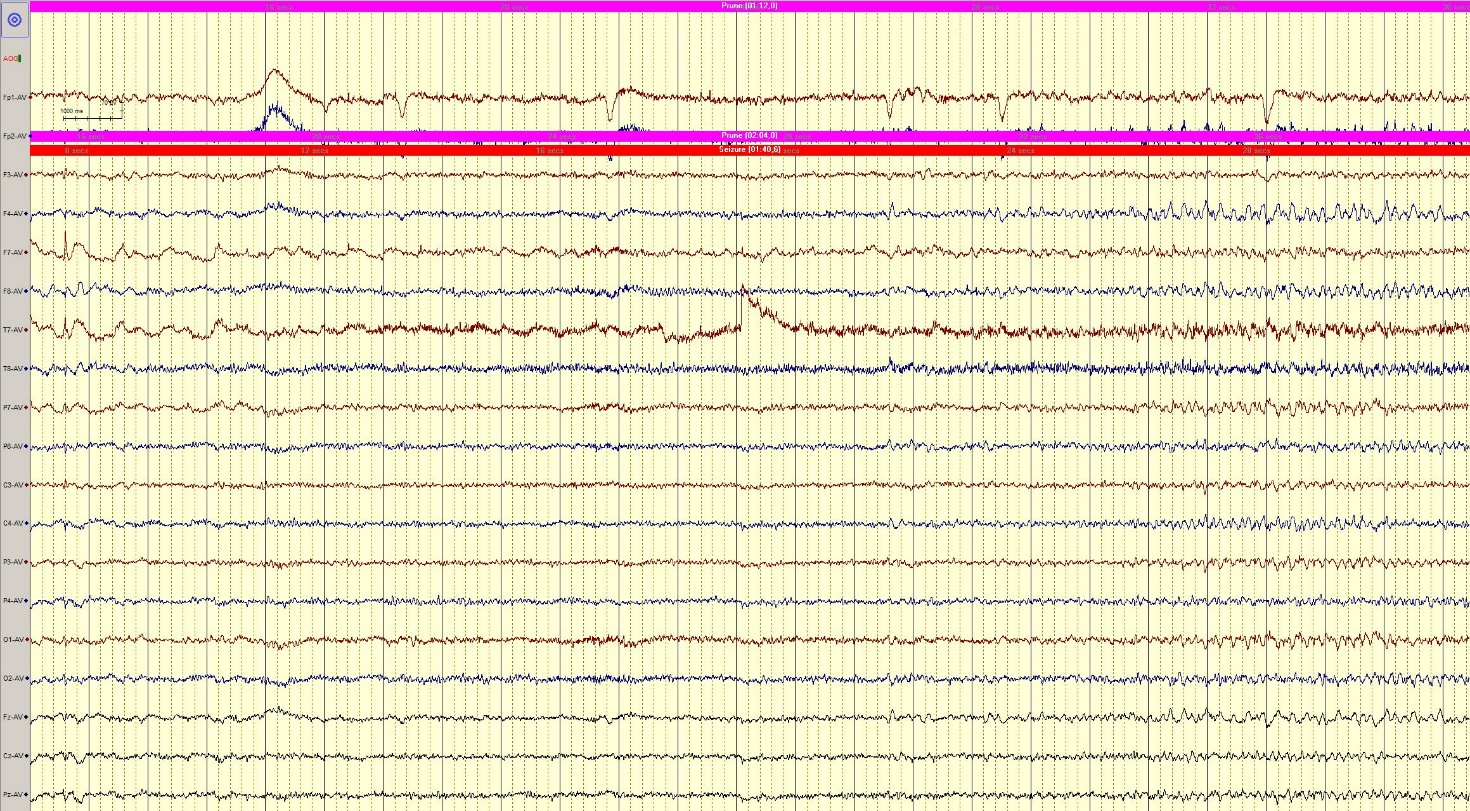


During the trial, she recorded the following paper seizure log:


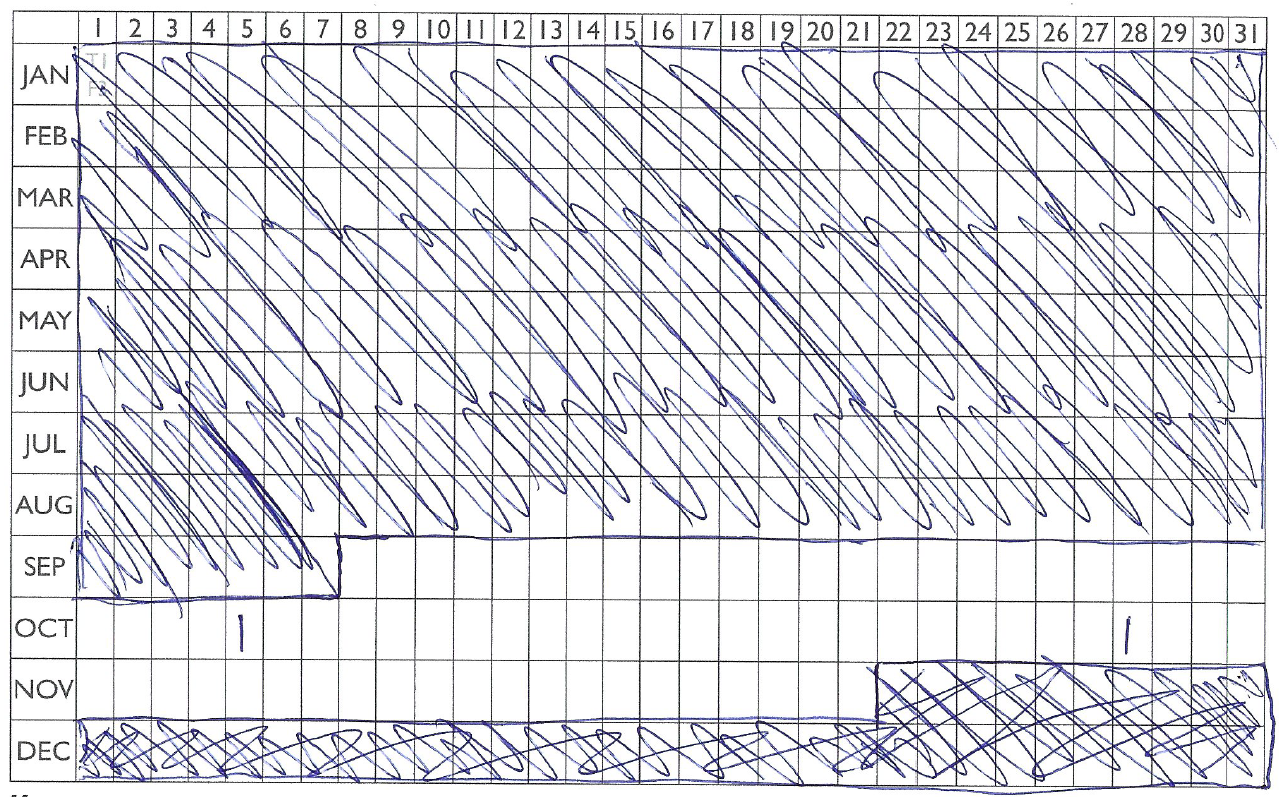

Supplement: Supplementary file 2 [file Data_Sheet_2.DOCX]
