# Supplementary material for: Expert Perspective: Who May Benefit Most From the New Ultra Long-Term Subcutaneous EEG Monitoring?
Source: Front Neurol. 2022 Jan 20;12:817733. doi: 10.3389/fneur.2021.817733 (PMC8810530; doi:10.3389/fneur.2021.817733)

**Clinical vignette 2 - LBO**

LBO had seizure onset at age 3, described as short seizures with impaired awareness although precise characteristics at time of onset are unknown. She was found to have a pleomorphic xanthoastrocytoma in the left temporal lobe at age 4. She underwent resective temporal lobe surgery at age 5, and again at age 9 and 13, while being treated with levetiracetam. She was tapered off AEDs at age 13, and was reportedly seizure free for the next 11 years.

At age 24, LBO began experiencing the following semiology:

1. Events with speech arrest. LBO was able to hear other people talking to her, but not understand their words. At the same time, she was unable to produce any words herself, but she was able to communicate non-verbally. Writing was also impaired. She reported 3-5 events per week.

Her neurological examination was normal, including memory. A 25-channel EEG (including sleep) showed 3-5 Hz activity almost continually over the left pre- and midtemporal regions with the occasional sharp transient in the same areas. 1,5T MRI with intravenous contrast showed sequelae from the prior surgeries in the left temporal region.

**
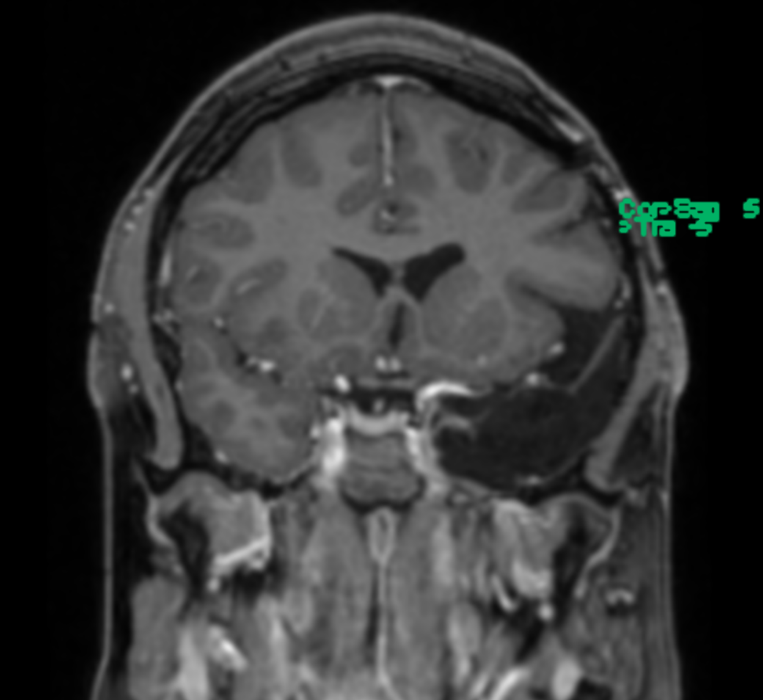

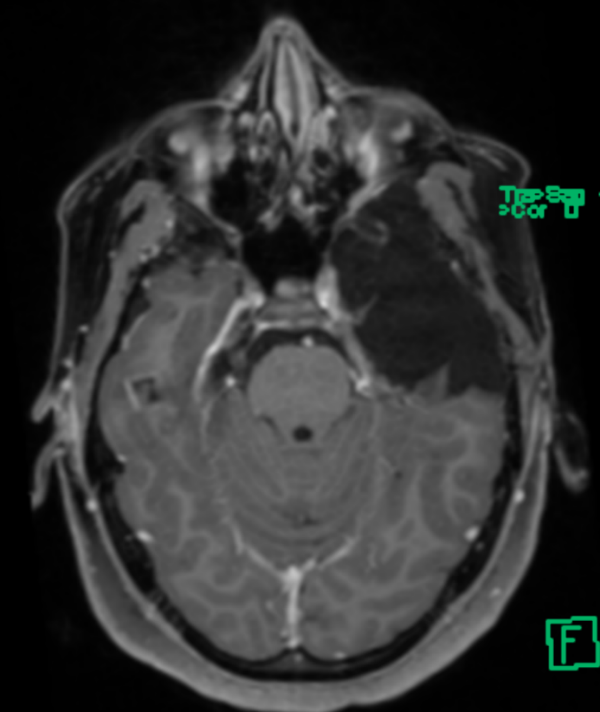
**

Due to an association between events and emotional stress, nonepileptic spells were considered in addition to electrical seizures. Therefore, she underwent two days of video-EEG, during which no clinical or electrographic events were captured. After ongoing events at home, the patient was readmitted for video-EEG monitoring and this time epileptic seizures arising from the left temporal region were captured, with the aforementioned clinical semiology. All three of the symptomatic events described by LBO were electrographic seizures, and LBO identified all of her electrographic seizures during the admission.

Common Average Montage

**
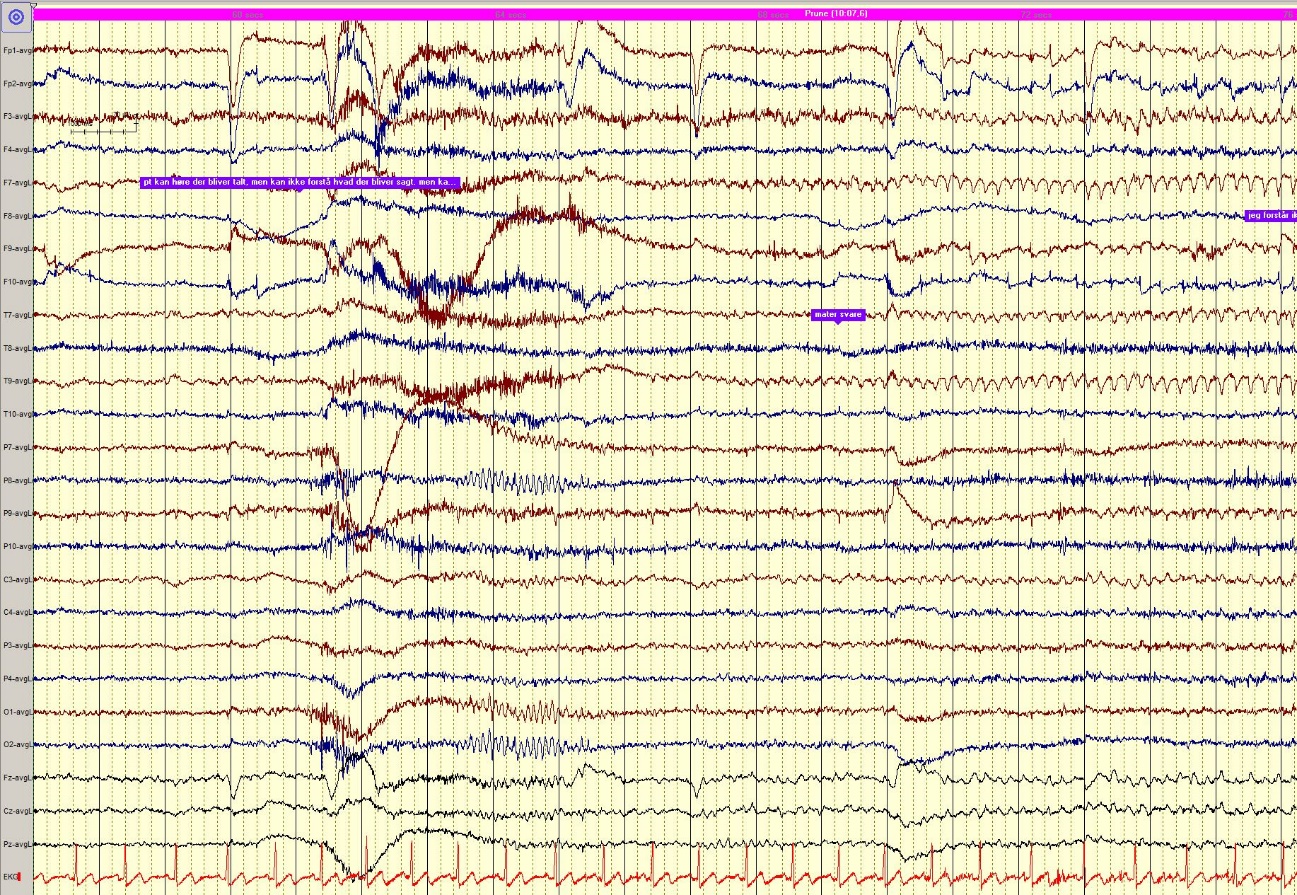
**

Longitudinal Bipolar Montage

**
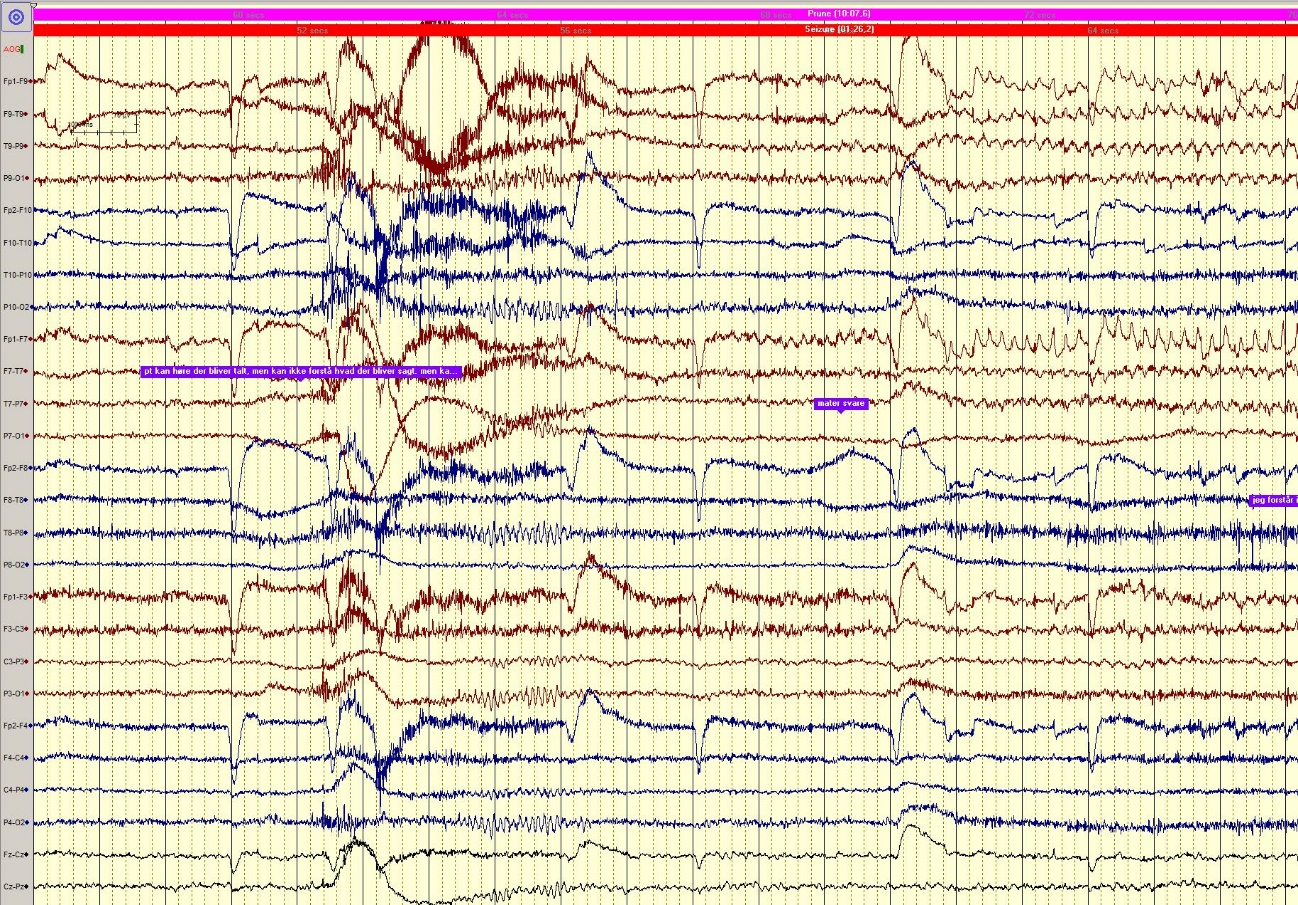
**

Antiepileptic therapy was recommended but LBO declined. She did, however, opt to participate in the UNEEG 24/7 subcutaneous EEG trial.

During the trial, she recorded the following paper seizure log:


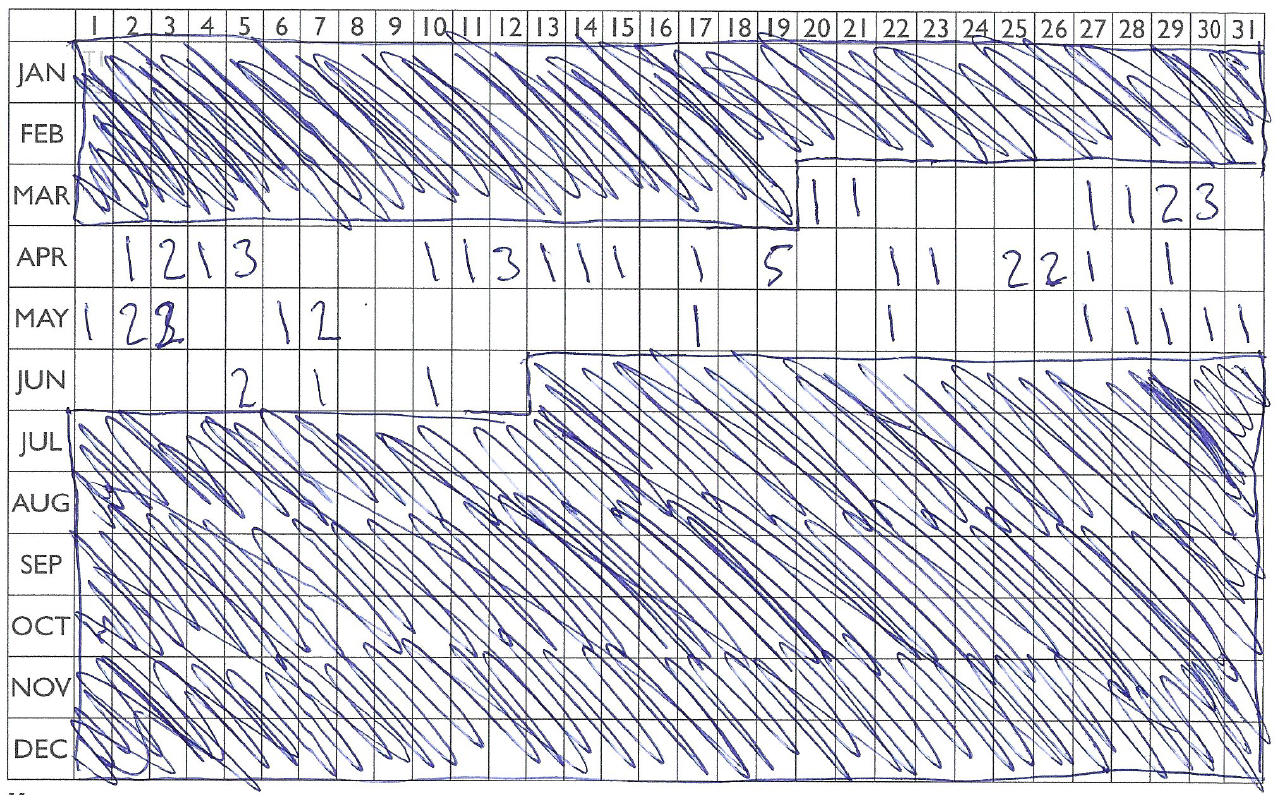

Supplement: Supplementary file 3 [file Data_Sheet_3.DOCX]
