# Supplementary material for: Expert Perspective: Who May Benefit Most From the New Ultra Long-Term Subcutaneous EEG Monitoring?
Source: Front Neurol. 2022 Jan 20;12:817733. doi: 10.3389/fneur.2021.817733 (PMC8810530; doi:10.3389/fneur.2021.817733)

# **Patient Details**

| **Name** | Patient 3 - SBN | **Sex** | Female |
| --- | --- | --- | --- |
| **Date of Birth**  **(age at study time)** | XXXX-XX-XX  33 years old | **Implant ID (placement)** | 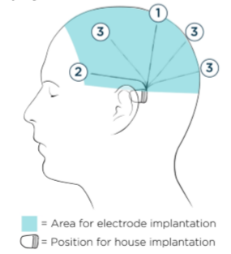XXXXXX  (Left 2) |
| **Indication for testing** | Part of clinical trial | **Treating Physician** | Sigge Weisdorf |

# **Recording and Report Details**

| Hours recorded | 1574 | Electrographic seizures: | 46 |  |
| --- | --- | --- | --- | --- |
| Usage total | 76% | Electrographic seizure rate | 0.53/day |  |
| Usage day (7-23) | 76% | Electrographic seizure rate  pr hour recorded | 0.70/day |  |
| Usage night (23-7) | 76% | Diary Entries | 118 |  |

# **Summary of the Findings**

| Registered 88 days of sqEEG. Raw EEG reviewed for all 46 electrographic seizures. Two seizures were evolving tonic-clonic. Rest start with burst of fast sharpened activity, evolving in morphology, not necessarily in frequency, followed by slow waves. All seizures out of sleep. |
| --- |

# **Diagnostic Significance**

| Epileptiform discharges and electrographic seizures |
| --- |

# **Clinical Comments**

| Moderate compliance – a bit lower later in recording. Tendency towards improvement of AED change. Seizures highly clustered on certain nights while self-report seems continuous without clustering. |
| --- |

# **Electrographic Seizure Details**

# Seizure Peridiocity


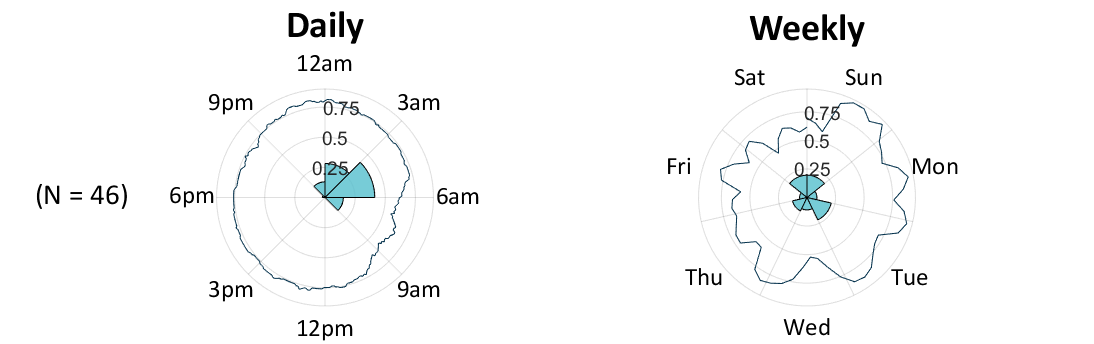


Usage (%)

# **Exemplar Electrographic Seizures**

Comment: Seizure with no motor phase


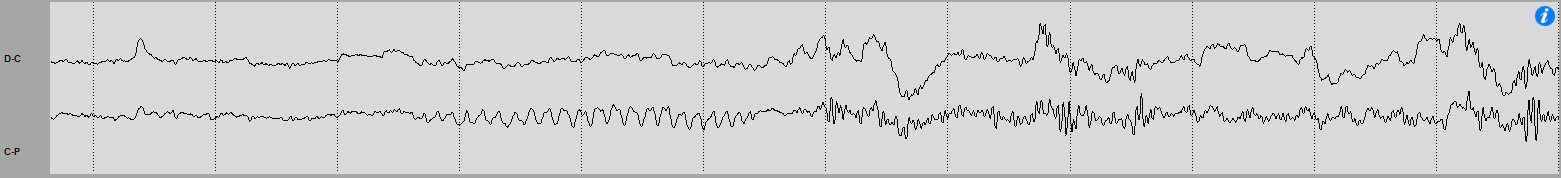


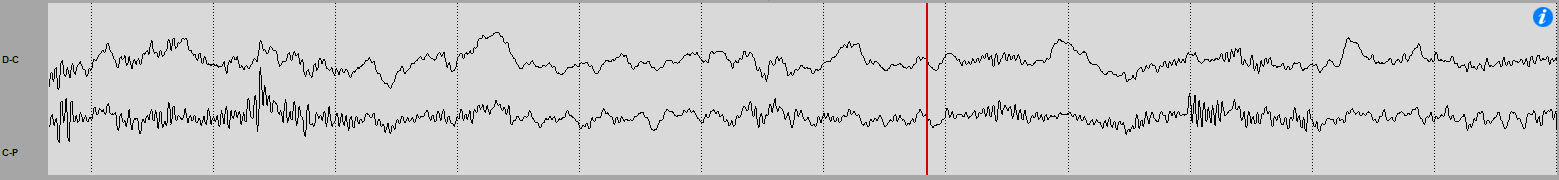


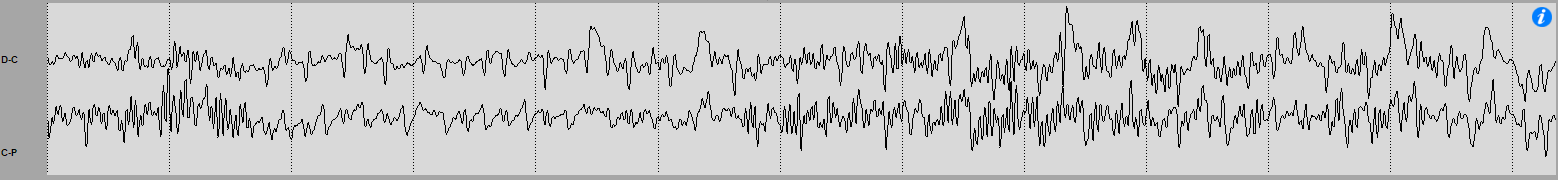


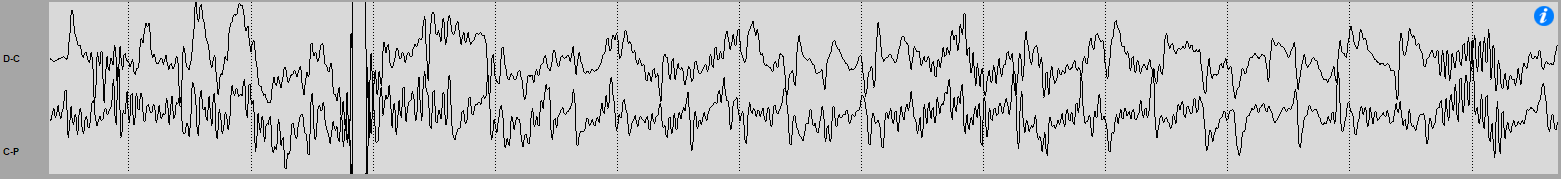


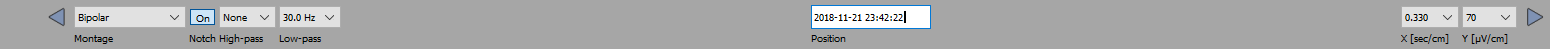

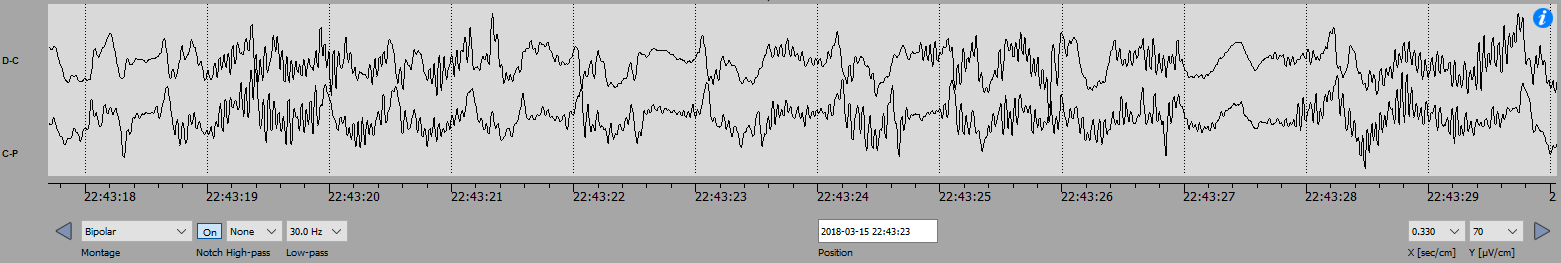


Comment: Evolving tonic-clonic seizure


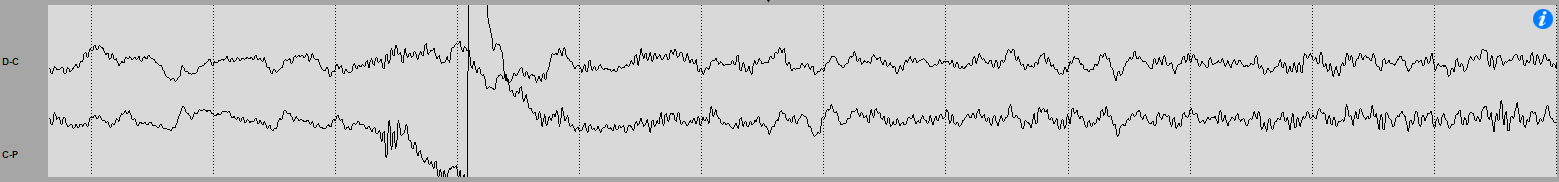


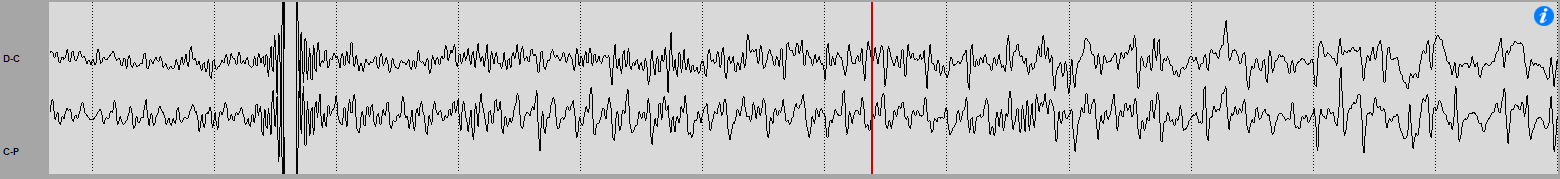


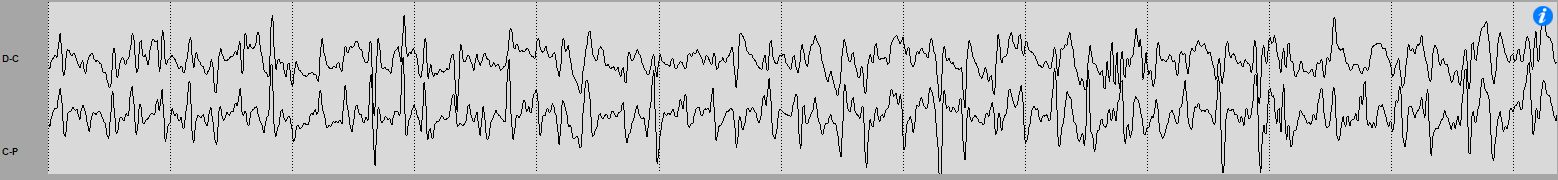


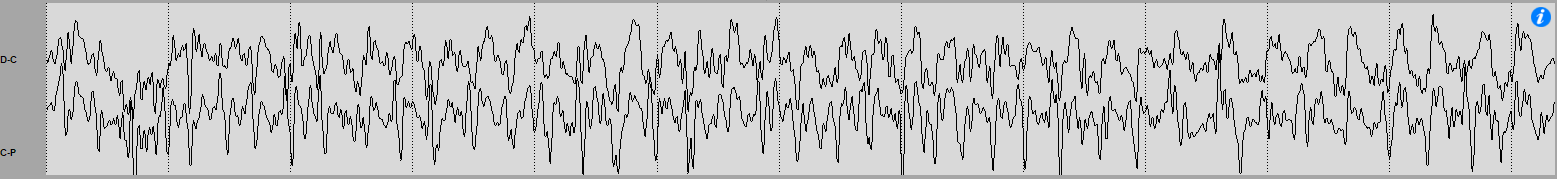


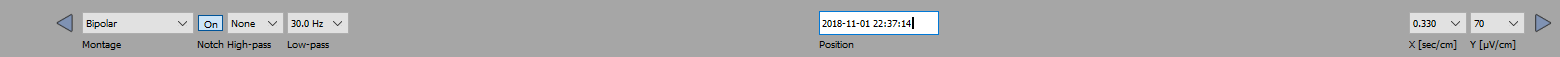

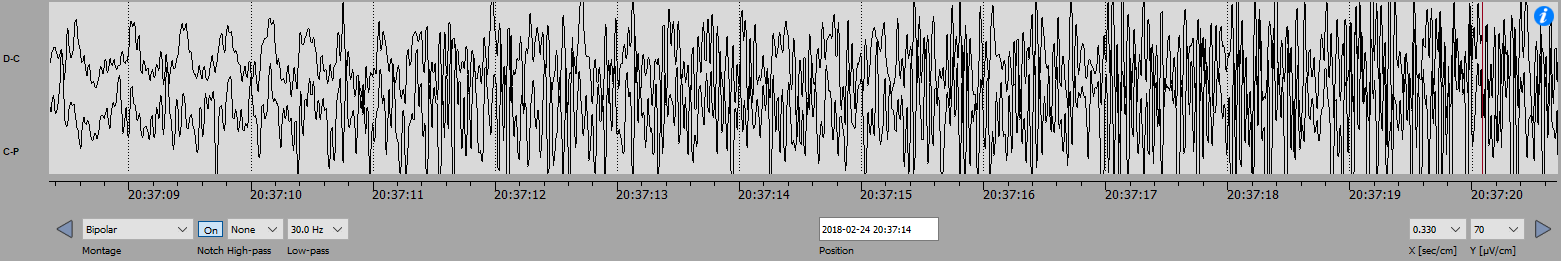

Supplement: Supplementary file 4 [file Data_Sheet_4.DOCX]
