# Supplementary material for: Expert Perspective: Who May Benefit Most From the New Ultra Long-Term Subcutaneous EEG Monitoring?
Source: Front Neurol. 2022 Jan 20;12:817733. doi: 10.3389/fneur.2021.817733 (PMC8810530; doi:10.3389/fneur.2021.817733)

# **Patient Details**

| **Name** | Patient 1 - J | **Sex** | Female |
| --- | --- | --- | --- |
| **Date of Birth**  **(age at study time)** | XXXX-XX-XX  43 years old | **Implant ID (placement)** | 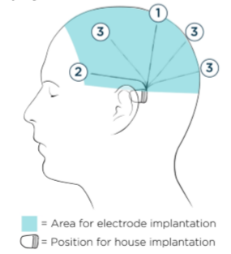XXXXXX  (Left 2) |
| **Indication for testing** | Part of clinical trial | **Treating Physician** | Sigge Weisdorf |

# **Recording and Report Details**

| Hours recorded | 1107 | Electrographic seizures: | 16 |  |
| --- | --- | --- | --- | --- |
| Usage total | 66% | Electrographic seizure rate | 0.23/day |  |
| Usage day (7-23) | 61% | Electrographic seizure rate  pr hour recorded | 0.35/day |  |
| Usage night (23-7) | 68% | Diary Entries | 2 |  |

# **Summary of the Findings**

| Registered 72 days of sqEEG. Raw EEG reviewed for all 16 electrographic seizures with clear paroxystic morphology. Two first seizures were evolving tonic-clonic. Rest were probably focal impaired awareness with an evolving theta-alpha rhythm. Seizure rate decreased during study correlated to lamotrigin dosage increase. |
| --- |

# **Diagnostic Significance**

| Epileptiform discharges and electrographic seizures |
| --- |

# **Clinical Comments**

| Moderate compliance – slightly better at night. Consider pt information. Good effect of change of AED. Very low overlap between reported (2) and electrographic seizures (16). |
| --- |

# **Electrographic Seizure Details**

# Seizure Peridiocity


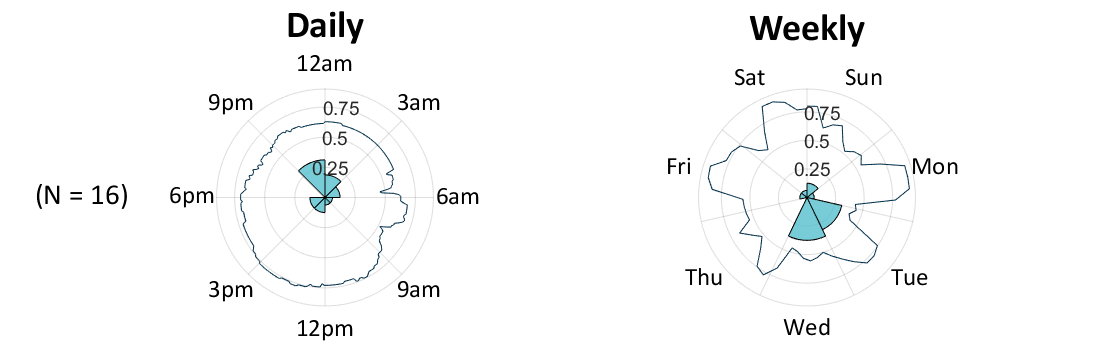


Usage (%)

# **Exemplar Electrographic Seizures**

Comment: Tonic-Clonic seizure. Duration: 1 min 34 sec


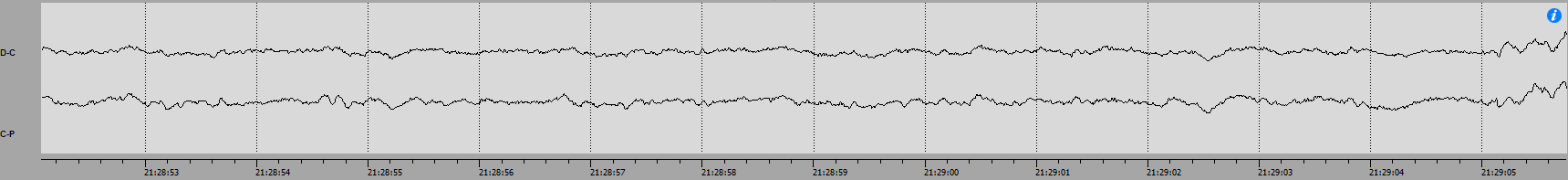


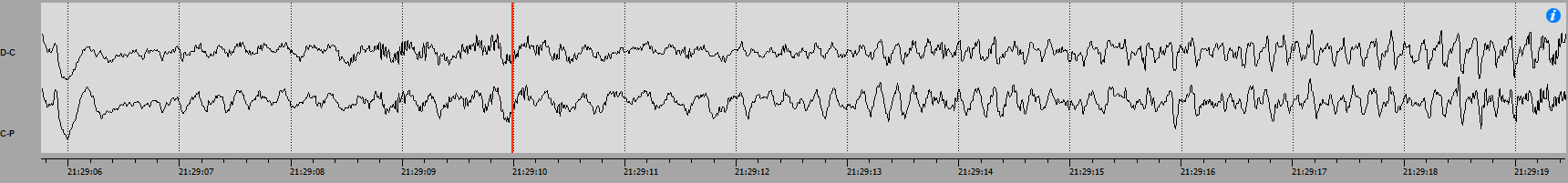


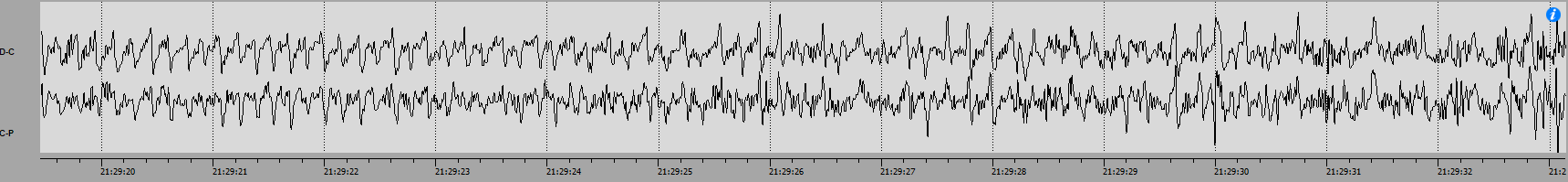


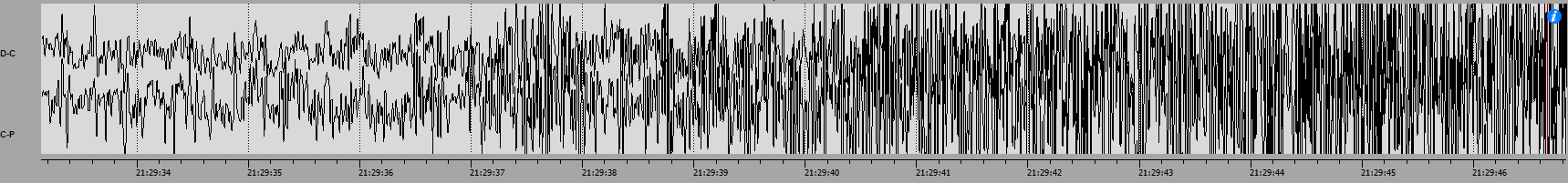


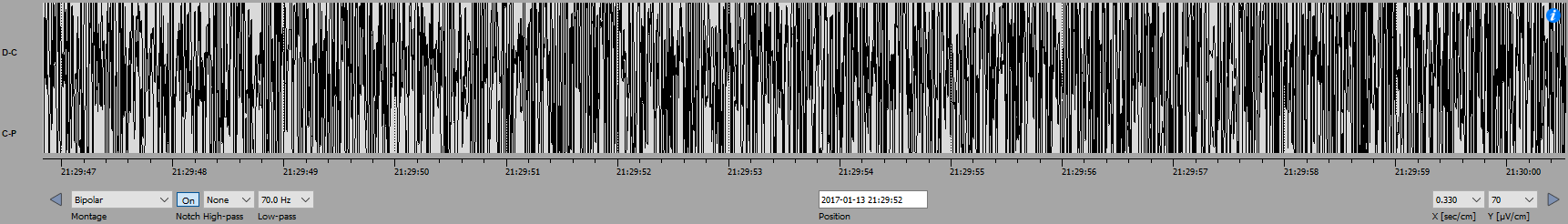


Comment: Focal impaired awareness seizure.


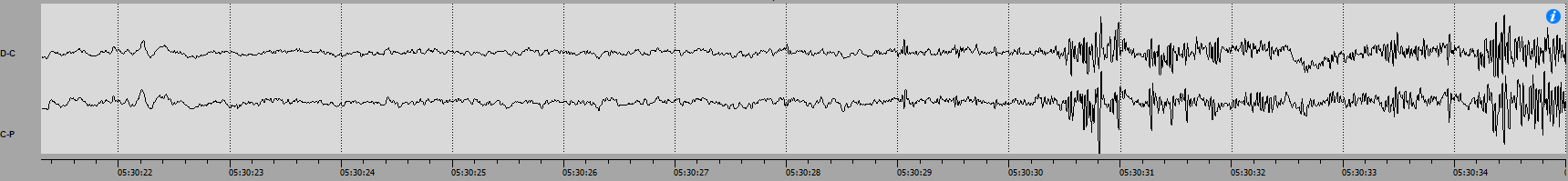


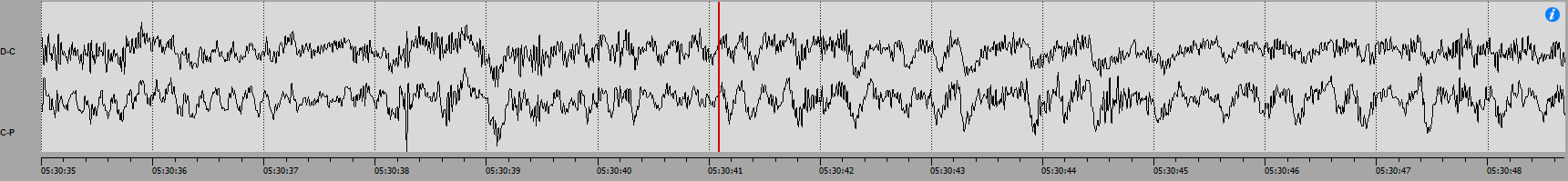


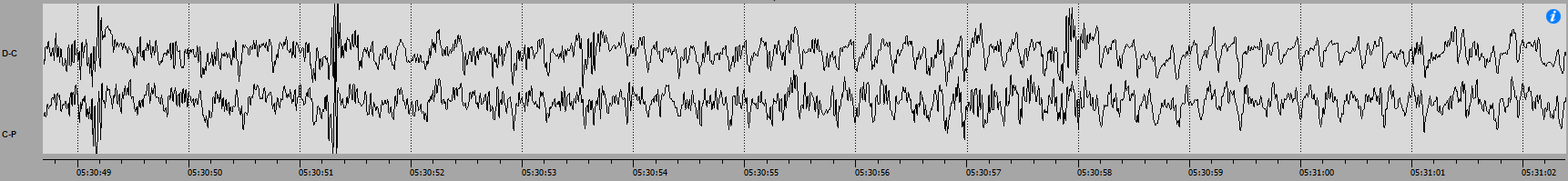


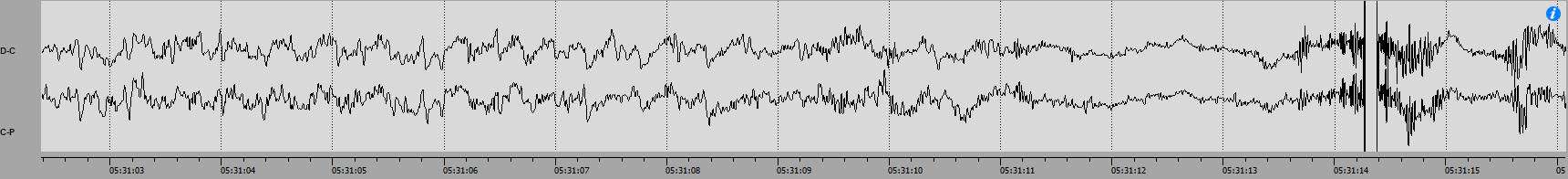


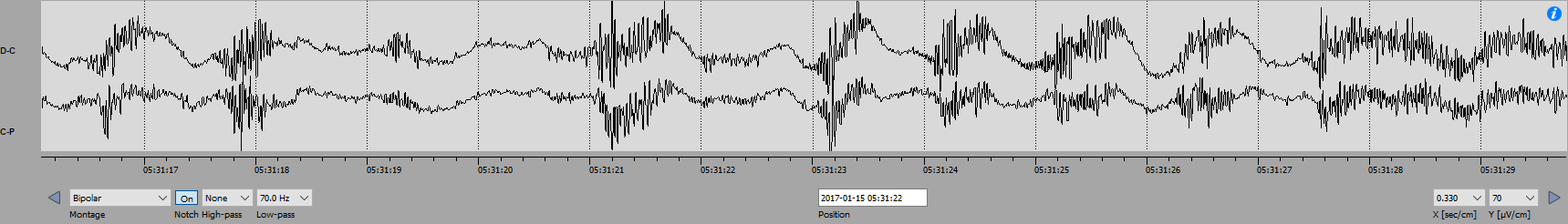

Supplement: Supplementary file 5 [file Data_Sheet_5.DOCX]
