# Supplementary material for: Expert Perspective: Who May Benefit Most From the New Ultra Long-Term Subcutaneous EEG Monitoring?
Source: Front Neurol. 2022 Jan 20;12:817733. doi: 10.3389/fneur.2021.817733 (PMC8810530; doi:10.3389/fneur.2021.817733)

# **Patient Details**

| **Name** | Patient 2 - LBO | **Sex** | Female |
| --- | --- | --- | --- |
| **Date of Birth**  **(age at study time)** | XXXX-XX-XX  27 years old | **Implant ID (placement)** | 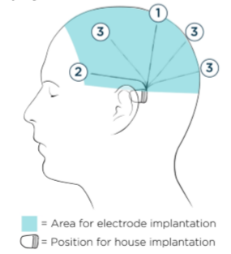XXXXXX  (Left 2) |
| **Indication for testing** | Part of clinical trial | **Treating Physician** | Sigge Weisdorf |

#
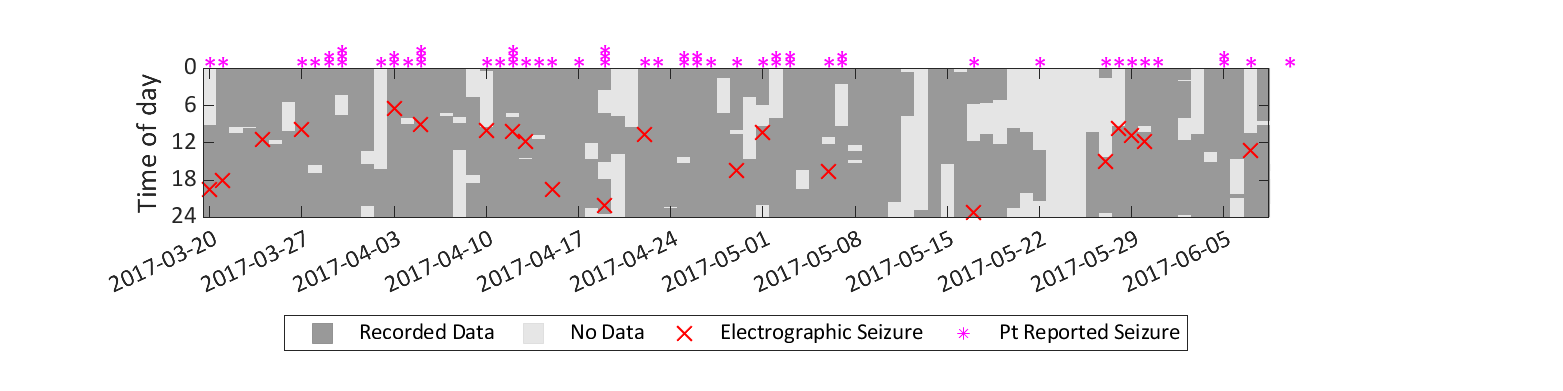
**Recording and Report Details**

Period 1

| Hours recorded | 1539 | Electrographic seizures: | 21 |  |
| --- | --- | --- | --- | --- |
| Usage total | 79% | Electrographic seizure rate | 0.26/day |  |
| Usage day (7-23) | 74% | Electrographic seizure rate  pr hour recorded | 0.33/day |  |
| Usage night (23-7) | 82% | Diary Entries | 56 |  |

# **Summary of the Findings**

| Registered 81 days of sqEEG. Raw EEG reviewed for all 21 electrographic seizures with clearly evolving rhythm under muscle artefact. Seizure rate fairly constant. |
| --- |

# **Diagnostic Significance**

| Epileptiform discharges and electrographic seizures |
| --- |

# **Clinical Comments**

| Good compliance except from longer continuous periods. Quite high overlap between reported (56) and detected seizures (21) although more than twice as many reported seizures. Half of seizures occurring between 9am-12pm. All seizures from awake. |
| --- |

# **Electrographic Seizure Details**

# Seizure Peridiocity


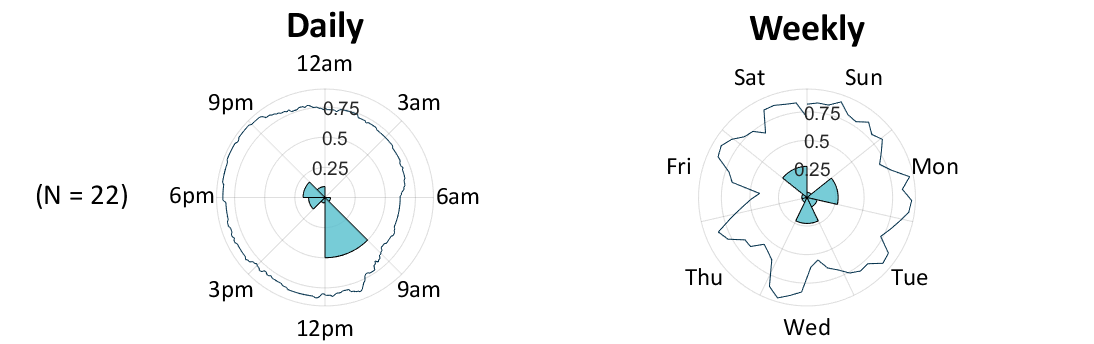


Usage (%)

# **Exemplar Electrographic Seizures**

Comment: Focal aware seizure


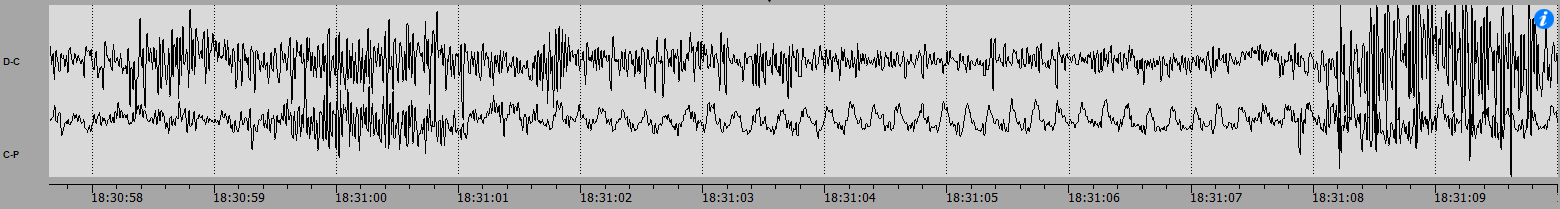


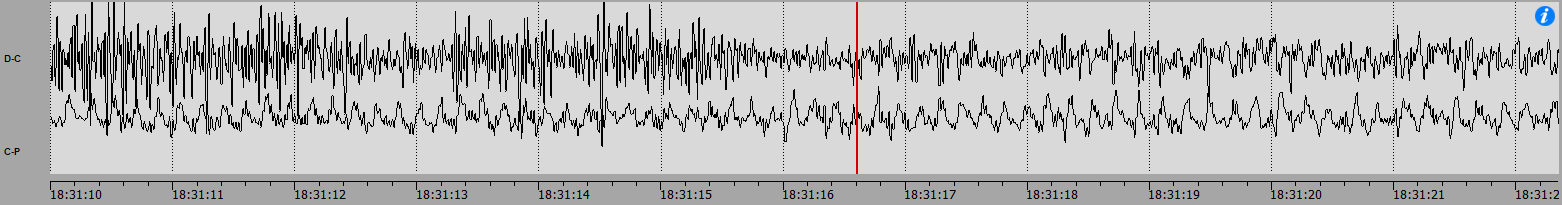


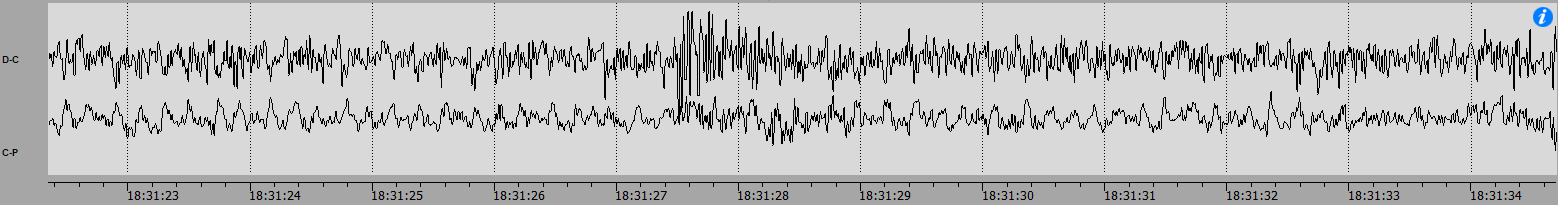


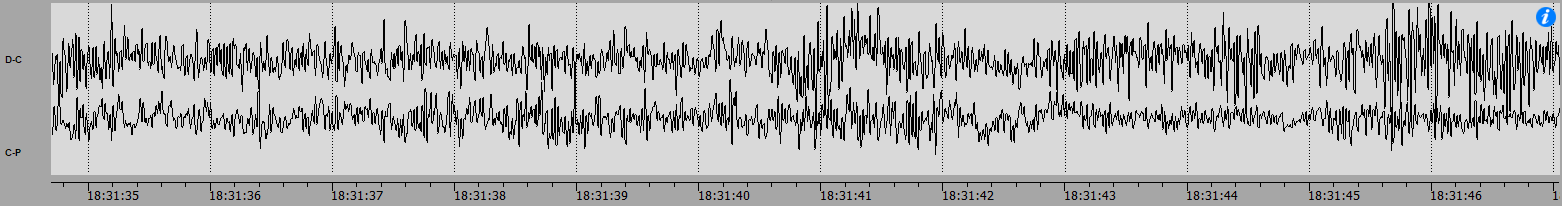


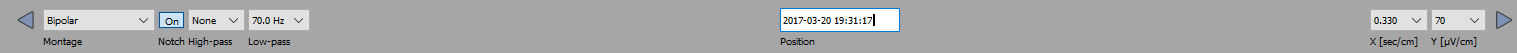

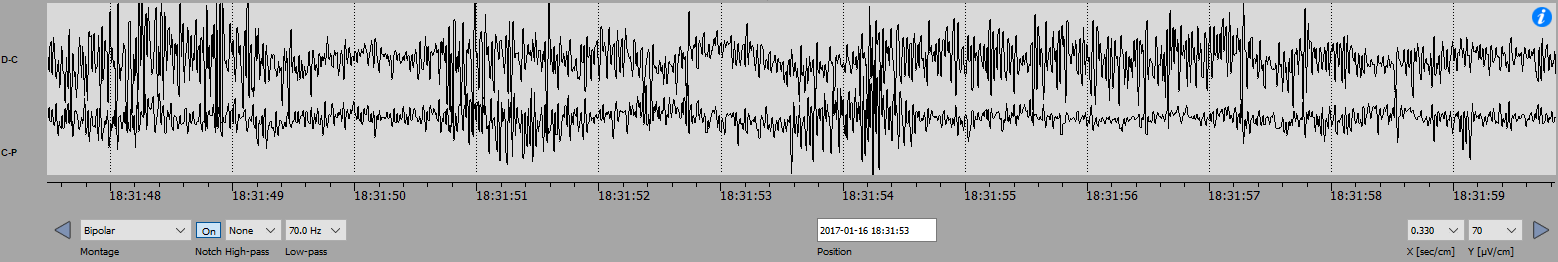


Comment: Focal aware seizure


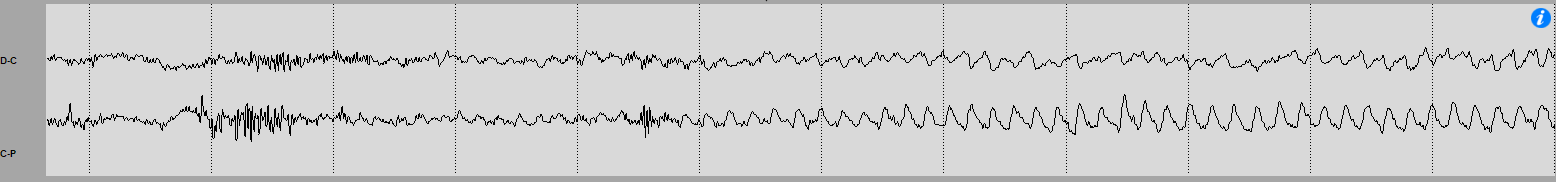


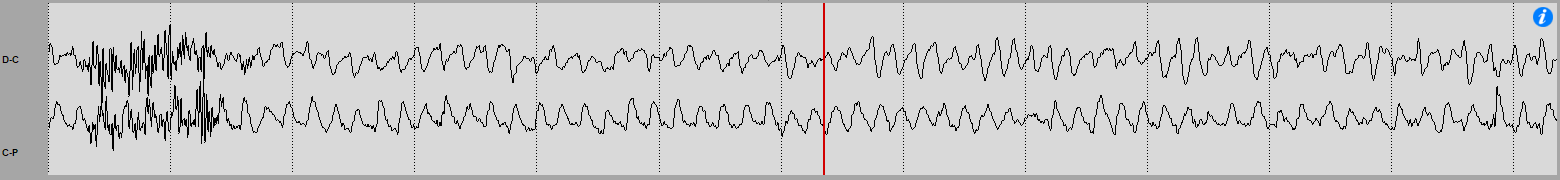


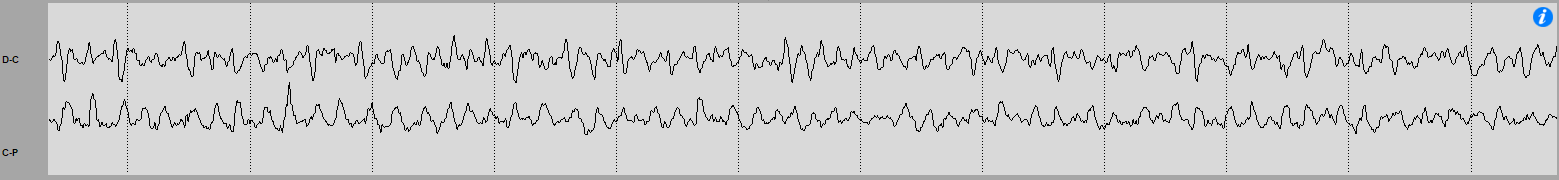


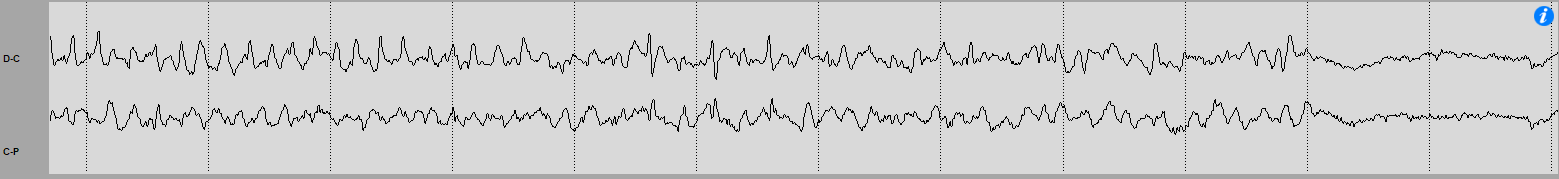


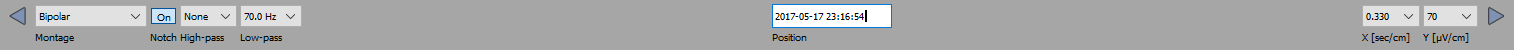

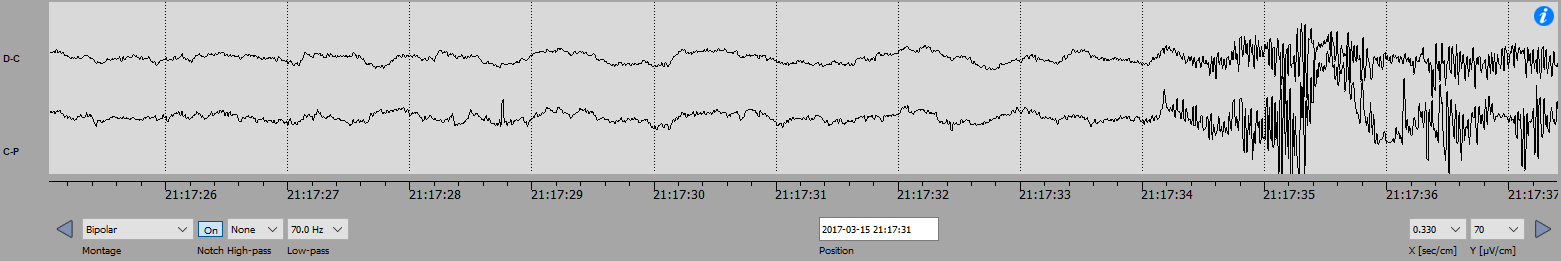

Supplement: Supplementary file 6 [file Data_Sheet_6.DOCX]
